# The three-dimensional structure of temporal framing in European adults: balancedness in past, future, and present time perspective

**DOI:** 10.3389/fpsyg.2026.1722605

**Published:** 2026-05-11

**Authors:** Hans-Joachim Lenz, Simon Forstmeier, Lukas Flöter, Nik Hulsmans

**Affiliations:** Department of Psychology, Developmental Psychology and Clinical Psychology of the Lifespan, University of Siegen, Siegen, Germany

**Keywords:** balancedness, Bayesian inference, lifespan, quality-of-life, time perspective, well-being

## Abstract

**Purpose:**

This study aims to provide evidence on how to best capture balancedness in temporal framing. Balancedness is assumed to be adaptive, predicting well-being and other indicators of mental health. Additionally, time perception is fundamental to human functioning, directing time perspectives (TP) toward past, present, and future orientations. There are various proposals to measure it, differing in both construct dimensionality and operationalization. Analyses are conducted in the broader context of well-being and quality of life, aiming for practical implications in lifespan psychology. This study compares the Balanced Time Perspective Scale (BTPS) with the modified BTPS, addressing methodological issues regarding whether two or three dimensions sufficiently capture time perspective, as well as quantifying explained model variance and its incremental gain from using two compound measures: Deviation from Balanced Time Perspective (DBTP) and Structural Equation Model Coefficient (SEM).

**Methods:**

This is an observational study using a cross-sectional design. Neither blinding nor random assignment of subjects was applied. Observations are uniformly distributed across 6 groups [18; 71+] for age and sex. Observations are evenly distributed for balancedness. The accuracy of the model is validated through five-fold cross-validation employing a training and test subset. Adherence to the requirements of the analytical models is ensured. Linear Discriminant Analyses are applied to infer evidence.

**Results:**

Balancedness in temporal framing has a three-factorial structure: The share of explained variance in a model that includes the present exceeds that of the past and future; hence, three time perspectives are better than two, i.e., mBTPS outperforms BTPS. DBTP and SEM, two comprehensive indicators for alternate application, further increase the explanatory power beyond the use of three TPs. The overall predictive ability of the model is excellent, ranking in the upper percentile of correct estimations. Specifically, evidence suggests that DBTP is more predictive of the absence of balancedness, whereas SEM better captures its presence. The experience of subjective well-being is, on average, higher in balanced observations. Balancedness does not interact with age or sex.

**Conclusion:**

Temporal balancedness has a three-factorial structure, best measured by a composite estimate of the past, future, and present time perspectives. The upper quartile of ratings in experienced Subjective Well-being (SWB) establishes a threshold with clinical implications.

## Introduction

### Time perspective

The construct of time perspective (TP), as understood in current psychological research, is based on the work of Lewin (1942) in the mid-20th century (Stolarski et al., 2018). Lewin described TP as “the totality of an individual’s views of his psychological future and psychological past existing at a given time” (Lewin, 1951, p. 75). While useful, this broader definition makes it difficult to differentiate TP from other psychological constructs such as *mental time travel* or *intertemporal choices* (Stolarski et al., 2018). Zimbardo and Boyd (1999) provided a more specific definition, describing TP as a subconscious process that organizes personal and social experiences into temporal categories or contexts. This process allows individuals to bring order, meaning, and coherence to their life experiences. Humans can shift their TP among past, present, and future biographical points in their lives. Some research conceptualizes TP as an orientation toward the past and future (Webster, 2011), while other models also include the present (Vowinckel et al., 2017; Zimbardo and Boyd, 1999). A past TP refers to how individuals think about their past and the meaning they assign to past experiences (Webster, 2011; Zimbardo and Boyd, 1999). For example, whether they reminisce about the past as a resource or ruminate on losses, i.e., redemptive versus contaminative framing (Cappeliez and O’Rourke, 2006; McAdams et al., 2001; Vowinckel et al., 2017; Webster, 2011). The present TP describes a focus on the current moment and the experience of the here and now (Burzynska and Stolarski, 2020; Prezpiorka and Sobol-Kwapinska, 2021; Vowinckel et al., 2017). Lastly, a future TP represents the temporal orientation toward the future and anticipated goals (Simons et al., 2004; Webster, 2011; Zimbardo and Boyd, 1999). It also encompasses motivation, anticipation, goal setting, and goal achievement (Stolarski et al., 2018; Zimbardo and Boyd, 1999). In this conceptual framework, some items of the Zimbardo Time Perspective Index (ZTPI) measure the future perspective in terms of time management, while Webster’s (2011) alternative Balanced Time Perspective Scale (BTPS) features both affective and cognitive facets in each item (Vowinckel et al., 2017).

#### Time perspective and well-being

TP is an important variable to consider in the context of psychological research because it is linked to several different psychological constructs, many of which are associated with quality of life, health, and well-being. A positive orientation toward the past TP is associated with increased well-being (Diaconu-Gherasim et al., 2021) and positive affect, whereas a negative orientation toward the past is linked to decreased well-being (Boniwell et al., 2010; Desmyter and De Raedt, 2012). Additionally, a positive past orientation in autobiographical memory is connected to resources (Ely and Mercurio, 2011), emotional intelligence (Stolarski et al., 2011), and self-esteem (Anagnostopoulos and Griva, 2012). A strong focus on the present TP is associated with living in the moment, seeking immediate pleasure, and yielding to temptations (Burzynska and Stolarski, 2020). However, these findings relate to studies using the Zimbardo Time Perspective Inventory (ZTPI; Zimbardo and Boyd, 1999), which focuses on the here-and-now experience (Fieulaine and Martinez, 2010; Keough et al., 1999). Positive associations with mindfulness, flow experiences, and improved mental health have been found with the present TP in the modified Balanced Time Perspective Scale (mBTPS; Vowinckel et al., 2017). Additionally, research highlights positive correlations with well-being (Przepiorka and Sobol-Kwapinska, 2021; Stolarski et al., 2018; Vowinckel et al., 2017), increased mood (Stolarski et al., 2014), emotional intelligence (Stolarski et al., 2011), extraversion, and openness to new experiences (Stolarski et al., 2018). A positive future TP orientation has been linked to better health-conscious behaviors such as lower substance use (alcohol and tobacco) or increased seatbelt use (Daugherty and Brase, 2010), attending preventive cancer screenings (Roncancio et al., 2014), and smoking cessation (Kovač and Rise, 2007). Additional correlations include reduced distress following traumatic events (Holman and Silver, 1998, 2005), reduced procrastination (Ferrari and Díaz-Morales, 2007), higher income and educational attainment (Shores and Scott, 2007), greater capacity for delayed gratification (Daugherty and Brase, 2010), and well-being (Vowinckel et al., 2017).

Overall, research shows individual TPs to be correlated with positive constructs such as health-conscious behavior (Daugherty and Brase, 2010) and increased resilience (Indirasari et al., 2019). However, a dominant focus on one individual TP to the exclusion of others can lead to negative consequences (Boniwell et al., 2010). The positive effect of a specific TP also seems to depend on the situational context. For example, a future TP is particularly useful in the context of goal attainment, possibly due to its negative association with procrastination (Boniwell et al., 2010; Ferrari and Díaz-Morales, 2007). Therefore, Zimbardo and Boyd (1999) looked beyond individual TPs and described the idea of a *Balanced Time Perspective* (BTP).

#### Balanced Time Perspective

Zimbardo and Boyd (1999) conceptualized BTP as the adaptive ability to flexibly switch between past, present, and future TP and utilize them in a situationally appropriate manner. Boniwell and Zimbardo (2004) also emphasized flexible use and situational adequacy as central aspects of BTP. In an optimally balanced TP, the individual TPs of past, present, and future function together flexibly (Zimbardo, 2002). A high BTP allows for adaptive and functional behavior without being rigid or stuck in a single TP (Boniwell and Zimbardo, 2004; Zimbardo and Boyd, 1999). Following an initial empirical examination of different TP profiles (balanced vs. unbalanced) (Drake et al., 2008; Zhang et al., 2013), multiple studies have been conducted on BTP, particularly due to its broad informational value (Stolarski et al., 2018, 2020). A high BTP is correlated with several measures of well-being and markers of physiological and psychological health (Boniwell and Zimbardo, 2004; Oyanadel and Buela-Casal, 2014). For example, associations have been found with sleep quality (Rönnlund and Carelli, 2018), health-related quality of life (Oyanadel and Buela-Casal, 2014), and symptoms of anxiety, obsessive-compulsive disorders, depression, stress, and substance abuse (Stolarski et al., 2020). Additionally, a high BTP is correlated with better adaptation following potentially traumatic experiences (Tomich and Tolich, 2021), which could explain the association between a high BTP and a lowered risk for PTSD (Stolarski and Cyniak-Cieciura, 2016). BTP has been shown to be more strongly correlated with measures of well-being than individual TPs (Diaconu-Gherasim et al., 2021). In some studies, it explained up to 40% of the variance in measures of well-being (Stolarski et al., 2020; Zhang et al., 2013). Therefore, the degree of balance appears to be an explanatory variable for risk factors of physiological and psychological illness.

TP’s impact on well-being and health is likely mediated by decision-making and chosen behavior (Boniwell, 2005; Lennings, 1996). A two-path model has been formulated to explain the effect of BTP on well-being (Chen et al., 2021), with both an indirect and a direct path (Cunningham et al., 2015). The indirect path, also referred to as the bottom–up path, accounts for the effect of individual TPs on an individual’s behavior that improves their life circumstances and, in turn, their well-being (Cunningham et al., 2015). TP is associated with experience, the evaluation of past and present events, and the preference for future goals; therefore, it impacts behavioral outcomes (Cummins et al., 2019). Due to the recursive nature of choice and overall life quality, the mutual impact of living conditions, habits, and well-being is bidirectional; thus, influence can be exerted in either direction, with both top–down and bottom–up pathways possible (Burzynska and Stolarski, 2020; Schimmack et al., 2002). The former describes a direct path in which the mental representation of a given context impacts life quality and well-being, whereas the latter relates to an indirect effect in which behavioral choices build habits that shape life quality. However, adaptation can be either restrictive or flexible, being supportive or dysfunctional. Evidence links TP to a broad range of mental and health-related outcomes, encompassing risky or health-promoting behavior, addiction, diet, gratitude, mindfulness, flow, and the experience of joy or savoring (Benowitz, 1992; Boniwell et al., 2010; Burzynska and Stolarski, 2020; Cunningham et al., 2015; Roglic, 2016; Rothspan and Read, 1996; Schimmack et al., 2002; Swan and Lessov-Schlaggar, 2007). Additionally, the mental anticipation of one’s own future has a direct effect on well-being (Holman and Silver, 2005), and present TP is associated with positive affect and joy, which can also have a direct impact on well-being (Stolarski et al., 2014). Overall, past TP primarily impacts well-being via the direct path of the immediate interpretation of past events. In contrast, both present and future TPs have a more pronounced effect through both paths (Cunningham et al., 2015). While the significant effects of a BTP have strong empirical support, there is ongoing debate regarding the assessment of BTP.

Moreover, future orientation in temporal framing follows usual trends that, in accordance with socioemotional selectivity (Carstensen, 1995, 2006; Carstensen et al., 1999), describe a positivity effect in aging, whereby individuals exert executive control over selective goal setting and distinct attention to actively downsize exhausting states of negative valence (Reed and Carstensen, 2012).

### Measures of (balanced) time perspective

Reliable and valid psychometric instruments are required to adequately assess BTP’s impact on well-being and the etiology of physical and mental health issues (Vowinckel et al., 2017; Webster, 2011; Zimbardo and Boyd, 1999). Additionally, past research has utilized different methods of aggregating subscales into an overall BTP score, which affects the reliability of the measurements (Jankowski et al., 2020; Zhang et al., 2013). In previous research, the ZTPI (Zimbardo and Boyd, 1999) has established itself as the most widely studied and utilized questionnaire for assessing TPs and BTP (Laureiro-Martinez et al., 2017; Stolarski et al., 2018). The ZTPI contains 56 items that can be divided into five subscales: past-negative (PN), past-positive (PP), present-fatalistic (PF), present-hedonistic (PH), and future (F). These scales measure temporal orientation as a cognitive–emotional representation of time (Boniwell et al., 2010). However, discussions about the instrument indicate that the evaluative component is not distinct across all scales and that some items of the future scale capture time management rather than affective concerns (Vowinckel et al., 2017). Nevertheless, the instrument shows good test–retest reliability of *r* = 0.70 to 0.80 over a 4-week gap (Zimbardo and Boyd, 1999). Additionally, it demonstrates good validity (Boniwell et al., 2010; Zimbardo and Boyd, 1999), as indicated by significant correlations with measures of mental health (*r* = −0.41 to 0.38; Vowinckel et al., 2017) and psychological strengths such as vitality, resilience, and hope (*r* = −0.47 to 0.37; Davis and Cernas Ortiz, 2017).

More recently, the mBTPS (Vowinckel et al., 2017), an expansion of the Balanced Time Perspective Scale (BTPS; Webster, 2011), was developed to include an assessment of the present TP. Both scales consist of items that consistently feature an affective component (Vowinckel et al., 2017; Webster, 2011). The mBTPS consists of 38 items divided into three subscales or factors. The past and future subscales contain 14 items each, while the subscale for the present TP has 10 items. Reliability for all three scales ranges from *α* = 0.88–0.92 (Vowinckel et al., 2017; Webster, 2011), which falls within a good to excellent range (Cronbach, 1951; Zinbarg et al., 2005, 2006). Regarding validity, the mBTPS correlates with several measures of mental health to varying degrees (*r* = 0.30–0.56; Vowinckel et al., 2017). The inclusion of the subscale for the present TP increases the explained variance in mental health measures by almost 11% when compared with the original BTPS (Vowinckel et al., 2017). Additionally, the present subscale shows only a weak to mild correlation with the factors of past and future. However, past and future TP have a moderate to strong correlation with each other (Vowinckel et al., 2017).

The ZTPI and mBTPS differ in a few significant ways related to the decomposition of mental time into the representation of cognitive and affective facets. The mBTPS assesses only the positive orientation of past TP, while the ZTPI also includes a negative orientation. As expected, there is a strong overlap between the subscales of the questionnaires that assess positive past TP, but less so between the ZTPI’s negative past subscale and the mBTPS past subscale (Vowinckel et al., 2017). Hence, the ZTPI and (m)BTPS differ in how they conceptualize cognition and affect in temporal framing. While the ZTPI assesses both dimensions on different scales, each item in the (m)BTPS jointly measures the cognitive and affective components of a respective time perspective. To address the external criterion validity of these facets, this study uses two measures: the Satisfaction with Life Scale (SWLS), which assesses the cognitive correlate of overall life quality, and the Short Scale of General Life Satisfaction (L-1), which is sensitive to capturing both cognitive and emotional evaluations (Beierlein et al., 2015; Cummins et al., 2019; Diener et al., 1985; Janke and Glöckner-Rist, 2014). Furthermore, evidence supports that selective goal attention, as conceptualized by the positivity effect or socio-emotional selectivity (Carstensen, 2006; Reed and Carstensen, 2012), serves well-being.

Another key difference is the varying conceptualization of present TP. The mBTPS subscale was derived from 10 items from the Present-Eudaimonic Scale (Vowinckel et al., 2017) and is based on the concept of eudaimonia, whereas the ZTPI’s subscale is based on the concept of hedonism (Huta and Waterman, 2014). This difference affects the association with well-being, as well-being is often divided into two perspectives: subjective (hedonic) well-being and eudaimonic well-being (Eid, 2021; Martela and Sheldon, 2019). Just as hedonism reflects subjective well-being, eudaimonia is an important aspect of well-being (Eid, 2021; Vowinckel et al., 2017). Eudaimonia encompasses aspects such as personal growth, meaning in life, self-realization, and authenticity (Ryan and Deci, 2001; Huta and Waterman, 2014). Hedonism reflects aspects such as pleasure, joy, and the absence of suffering (Huta and Waterman, 2014). In research on BTP, subjective well-being usually functions as the criterion variable (Diaconu-Gherasim et al., 2021). Subjective well-being consists of life satisfaction and positive/negative affect (Eid, 2021; Martela and Sheldon, 2019). Life satisfaction is generally considered a relatively stable trait, while affect is understood as a variable state (DeNeve and Cooper, 1998). Life satisfaction reflects a cognitive evaluation of one’s experiences and overall quality of life (Cummins et al., 2019; DeNeve and Cooper, 1998; Veenhoven, 2015).

The ZTPI also contains more items and factors, allowing for a more detailed assessment. However, it is less economical compared to the mBTPS due to its length (Vowinckel et al., 2017; Webster, 2011; Zimbardo and Boyd, 1999). Additionally, the ZTPI assesses both negative and positive orientations of past and present TPs, while the mBTPS only captures positive orientations (Vowinckel et al., 2017; Zimbardo and Boyd, 1999). Although the factors of the two scales correlate, the mBTPS shows a stronger association with mental health, particularly its subscale for the present TP (Vowinckel et al., 2017). Finally, the mBTPS was specifically designed for the assessment of BTP, whereas the ZTPI was not (Vowinckel et al., 2017).

#### How to balance time perspectives?

Beyond the differences between the questionnaires, several methods for calculating BTP have been proposed in past research (Stolarski et al., 2011; Jankowski et al., 2020; Vowinckel et al., 2017). Studies on the ZTPI have shown that the method used to aggregate individual TPs into a total BTP score can influence the interpretability of the results (Zhang et al., 2013).

The median-split approach has been applied to both the ZTPI and the mBTPS (Webster, 2011). By splitting the sample along the median, every participant can be assigned to either the low-scoring or the high-scoring group. As this can be done for each individual TP, different profile combinations can be distinguished. For example, four different combinations have been described for the BTPS with its two subscales: *time restrictive* (low past and future TP), *reminiscers* (high past and low future TP), *futurists* (low past and high future TP), and *time expansive* (high past and future TP) (Webster, 2011). The time expansive profile has been associated with increased well-being and happiness (Webster, 2011), as well as better mental health (Webster and Ma, 2013). However, approaches to measure BTP coexist, and evidence has been collected for both instruments, but the precedence of a particular metric is still undetermined.

Cluster analysis is a descriptive method that groups a large pool of data into homogeneous subgroups (Wirtz, 2022a). Hierarchical cluster analysis (HCA) combines data into increasingly larger groups in a stepwise process until all cases are grouped into one large cluster. Detailed descriptions of HCA have been provided in past research (Frades and Matthiesen, 2010; Köhn and Hubert, 2015). Prior studies have identified either four to five clusters (Boniwell et al., 2010) or a two-cluster solution (Zhang et al., 2013). Individuals in different clusters differ in their respective TPs and in measures of well-being such as life satisfaction. The clusters can be further evaluated according to their proximity to a BTP. However, like the median-split, HCA is dependent on the analyzed sample (Boniwell et al., 2010).

To address this limitation, an aggregation method introduced an approach that is independent of the analyzed sample (Wiberg et al., 2012). TPs are categorized based on predetermined and sample-independent reference values according to a certain ZTPI profile (Boniwell and Zimbardo, 2004). The mean for each TP subscale is calculated and then compared against the reference value. In a second step, overall BTP is assessed by counting how many means fall within the respective optimal range. Zero scores in the optimal ranges are considered to indicate a completely unbalanced profile, while all scores being optimal represent a perfectly balanced BTP.

Another sample-independent aggregation method is a distance metric (Stolarski et al., 2020), i.e., Deviation from Balanced Time Perspective (DBTP), which quantifies the Euclidean distance between the recommended and actual values of an observed TP. The reference values are based on a certain ZTPI profile, which is assumed to be optimal (Boniwell and Zimbardo, 2004). It is important to note that this property has not been supported by evidence but is rather argued to be balanced (Stolarski et al., 2020).

#### Covariates of Balanced Time Perspective and well-being

Past research has examined three potentially relevant sociodemographic covariates in the relationship between BTP and well-being: age, sex, and cultural context (Diaconu-Gherasim et al., 2021; Stolarski et al., 2020). Results regarding the effect of age have been heterogeneous. A meta-analysis from 2017 reported a positive correlation between age and the positive-past subscale of the ZTPI, but no association with future TP (Laureiro-Martinez et al., 2017), or found a negative association between future TP and age (Henry et al., 2017). A recent meta-analysis revealed a differentiated effect of age on measures of well-being (Diaconu-Gherasim et al., 2021). Specifically, BTP appears to be correlated with life satisfaction among younger individuals, whereas it rather covaries with global well-being in older persons. Evidence from our own study on BTP supports this finding, as adequate levels of well-being can be found across all age groups (Lenz et al., 2025). The literature does not support an effect of sex on BTP (Stolarski et al., 2020). In addition, although culture modulates a variety of psychological outcomes, no influence has been reported for future TP or BTP (Diaconu-Gherasim et al., 2021).

### Study purpose

The optimal calculation of an overall BTP remains a particularly important topic of research. While several studies have focused on aggregating the BTP with the ZTPI, other instruments such as mBTPS have received little to no attention. It therefore remains unclear whether the interpretability and predictive value of the BTP scores measured with the mBTPS can be improved by different aggregation methods. The aim of the present study was to compare the calculation method for the BTP proposed by the authors of the mBTPS with other potential calculation methods in terms of criterion validity. Aggregation methods from other measurement instruments (e.g., Jankowski et al., 2020; Stolarski et al., 2015; Zhang et al., 2013) were considered and incorporated into the current study to derive new potential calculation methods for the mBTPS. Additionally, a data-driven calculation approach based on a structural equation model (SEM) was included for comparison. Caution has been taken regarding the prior distribution of BTP, which inevitably varies according to the respective measure. Classification scenarios are sensitive to prevalence (Kuhn and Johnson, 2020), so the empirical proportions of balancedness between groups are expected to influence the empirical outcomes of the statistical model.

While balancedness is a robust indicator of well-being, evidence on an age-graded impact of temporal framing varies. The positivity effect describes selective goal attention as the ability of the elderly to actively downsize states of negative valence. The evidence from this study aims to clarify whether balancedness modulates this executive function. As balancedness is expected to enhance adaptivity, the level of subjective well-being may also serve as a clinically relevant cutoff value. The presented feature engineering procedure optimizes the compilation of candidate predictors while preventing the analytical model from overfitting the data. More important than merely discussing scale properties, it is proposed that good model accuracy largely depends on the sample prevalence, preventing prior knowledge from being propagated as a bias into the estimates.

## Methods

### Sample

The sample of the present study comprises *N* = 302 persons. Recruitment was carried out via an external service provider.^1^ Considering the existing literature, which reports medium effect sizes (e.g., Diaconu-Gherasim et al., 2021; Vowinckel et al., 2017), the sample size can be regarded as sufficient. No power analysis applied to sampling due to the exploratory nature of the study. The only eligibility criterion for participation was a minimum age of 18 years. In addition, restrictions on sex and age segmented the sample into six uniformly distributed classes.

Recruitment was carried out by the service provider, who invited individuals with the link to participate in the online study and ensured that the inclusion criteria and equal distribution were met. Data collection took place between 20.03.2022 and 28.03.2022 (soft launch on 16.03.2022, *n* = 30). The sample consists of a German panel of western European adults from various cultural backgrounds and encompasses a broad range of socioeconomic statuses; e.g., 56.00% are employed, 36.50% are retirees, and in terms of education, 13.20% completed secondary school (8 years) and 28.50% completed secondary school (10 years), 14.90% attended grammar school, 12.90% attended college of higher education, and 15.60% finished high school.

Missing values were handled through listwise deletion, and the pattern of missing data is consistent with missingness completely at random (MCAR). A total of *N* = 80 participants failed the quality check, which ensured the reliability of the responses through three items spread throughout the survey that had to be filled out according to the instructions, resulting in a final sample size of *N* = 302. For Subjective Well-being (SWB), a binomial logistic regression was non-significant [*χ*^2^ (1) = 0.122, *p* = 0.727], so missing values for that measure were substituted with the mean. Outliers were controlled for balancedness using Cook’s *D* (*statistical analyses;* Supplementary materials). Additionally, *n* = 26 cases with extreme values in processing time (+/− 3 × IQR) were inspected. Because explanatory analyses showed no systematic differences in outcomes, these observations remained in the dataset.

The final sample (*N* = 302) consisted of 151 (50.0%) females, 150 (49.7%) males, and 1 (0.3%) individual identifying as diverse. The mean age of the participants was *M* = 50.54 (*SD* = 17.49). In the lower age groups, females are predominant, while this trend reverses in the higher age groups [*χ*^2^ (5) = 19.18, *p* = 0.002] (Supplementary materials). As sex is not a covariate of balancedness, the unequal distribution can be disregarded at this point (Stolarski et al., 2020). While cultural background modulates the experience of well-being, no evidence exists regarding BTP (Diaconu-Gherasim et al., 2021), so this covariate is omitted from the analyses.

### Procedures and design

The study was approved by the ethics committee at the University of Siegen. In this correlational cohort study with a cross-sectional design on predictors of balancedness, subjects were not randomly assigned to a treatment and no blinding was involved; age and sex were equally distributed across 6 classes [18; 71+]. Random draws prepared for five-fold cross-validation, which was conducted on subsets in an 80:20 proportion for training and testing the model. Throughout, BTP prevalence is conditioned on even odds.

Participants accessed the study via a link and following informed consent, data were collected at one measurement point, and the questionnaires were presented in a fixed order. The average processing time was *M* = 23.93 min (Supplementary materials). Once all questionnaires were completed, participants were directed back to the external provider for payment.

Feature selection procedure, as detailed in the statistical analyses, operated on an initial set of measures that have been shown in the literature to covary with well-being, such as rumination, positivity, and executive functioning in selective attention and goal setting. Proof of internal consistency first assured overall scale quality. Subsequently, the resulting candidates were checked for eligibility in the analytical model (linear discriminant analysis, LDA) to ensure that each predictor met two criteria: it had to be both selective (*F*-statistic) and predictive (logistic regression) of balancedness.

### Research questions and general hypotheses

The study objective connects to the primary analyses of this research project. Most of the following hypotheses are exploratory due to the current state of knowledge in the scientific literature. Methodologically, linear discriminant analysis (LDA) will examine which model can best estimate balancedness, either in classifying or predicting purpose. It is expected that metric indices will outperform categorical predictors in both efficiency and accuracy. Evidence will be collected on whether the measurement of both balancedness and the psychological covariates of temporal framing requires the assessment of two or three time perspectives; thus,

Which predictor set is most appropriate for adequately classifying and predicting BTP? When ordering TP (past, present, future) according to their importance, what is their relative contribution to capturing BTP? When adding present TP, what is the incremental gain in explained variance compared to a model that comprises only past and future TP, i.e., mBTPS versus BTPS?

To what extent do metric indices, DBTP and SEM, outperform the use of TP scales in mBTPS or BTPS in classifying or predicting balancedness? The expectation is that both DBTP and SEM will yield predictions of higher accuracy.

Subjective well-being is expected to differ between balanced and unbalanced observations, with the balanced subsample reporting SWB in the upper quartile of average ratings (i.e., L-1 ≥ Q75; Cummins et al., 2019); similarly, adaptivity in temporal framing suggests that SWLS predicts balancedness.

Does the experience of well-being differ between subsamples of (un-)balanced observations? Is life quality predictive of levels of balancedness?

#### Materials

##### Modified Balanced Time Perspective Scale (mBTPS)

The mBTPS (Vowinckel et al., 2017) is based on the two subscales (past and future) of the BTPS (Webster, 2011), supplemented by a third subscale for the present time perspective. The instrument comprises a total of 38 items, which can be answered on a six-point scale (1 = never, 6 = always). The authors suggest using the DBTP coefficient to calculate a total BTP score (Vowinckel et al., 2017). The internal consistency of the subscales ranges from *α* = 0.88 (past and present) to *α* = 0.92 (future).

##### Satisfaction with Life Scale (SWLS)

The German version of the Satisfaction with Life Scale (Janke and Glöckner-Rist, 2012) is used in the present study to assess life quality. The SWLS measures the cognitive–evaluative component of well-being and contains five items that can be answered on a seven-point scale (1 = strongly disagree, 7 = strongly agree). The sum of all items forms the total scale value. The internal consistency is *α* = 0.92.

##### Short scale to measure general life satisfaction (L-1)

The L-1 (Beierlein et al., 2015) consists of one item that measures well-being in terms of global life satisfaction. This study asks “How satisfied are you with your life as a whole?” (adapted, original wording Cummins et al., 2019) Ratings can be given on a scale from 1 (dissatisfied) to 100 (satisfied). The test–retest reliability (6 weeks) is *r_tt_* = 0.67. In addition, values of *r* = 0.70–0.73 are reported for the correlation between the L-1 and the SWLS (Beierlein et al., 2015).

##### Patient Health Questionnaire-15 (PHQ15)

The PHQ15 (Kroenke et al., 2002) enables a dimensional assessment of the severity of somatic symptoms. Items 1–13 address various somatic complaints. A filter question precedes the subsequent symptom list: “How often do you feel bothered by the following somatic complaints in the last 4 weeks?”. Items 14 and 15 complement the symptoms referring to a somatic syndrome in the context of a depressive disorder, again preceded by “How often do you feel bothered by the following symptoms in the last 2 weeks?”. The first 13 items are answered on a three-point scale (0 = not bothered at all, 2 = bothered a lot), while items 14 and 15 can be answered on a four-point scale (0 = not at all, 3 = nearly every day). Summing the items of the two respective subscales yields two total scores. The internal consistency is *α* = 0.83.

##### Self-Control Scale (SCS-K-D)

The short from of the SCS in German language (Bertrams and Dickhäuser, 2009) is used to measure the dispositional self-control of the individuals. The short form comprises 13 items, which can be answered on a five-point scale (1 = not at all, 5 = very much). An internal consistency of *α* = 0.80 is reported.

##### Savoring Belief Inventory (SBI)

The SBI (Bryant, 2003) comprises 24 items, which are equally distributed across three factors. Each of these three factors (Anticipating, Savoring the Moment, Reminiscing) contains four positively and four negatively polarized items. The items are answered on a seven-point scale (1 = strongly disagree, 7 = strongly agree). An overall score across all items and a score per factor can be calculated. Negatively polarized items must be inverted. The internal consistency ranges from *α* = 0.68 to 0.94.

##### Prioritizing Positivity (PPQ)

The PPQ measures cognitive–emotional processes that express selective goal attention, “[…] the extent to which people seek out positive emotional experiences […]” (Catalino et al., 2014, p. 4). The questionnaire consists of 6 items, which can be answered on a nine-point scale (1 = strongly disagree, 9 = strongly agree). An internal consistency of *α* = 0.81 is reported.

##### Reminiscence Function Scale (RFS)

The RFS measures reminiscence in service of different psychological functions, comprising 43 items that can be answered on a six-point scale (1 = never, 6 = very frequently). The original version of the RFS (Webster, 1993, 1997, 2002, 2003) contains eight facets of reminiscence (Identity, Problem Solving, Death Preparation, Teach and Inform, Conversation, Bitterness Revival, Boredom Reduction, and Intimacy Maintenance). Combining the first two facets into one dimension (Identity/Problem Solving) supports a factorial structure of seven subscales (Robitaille et al., 2010; Washington, 2009; Webster, 1997). Overall, the evidence suggests a model of eight functions, clustered into three latent second-order factors: prosocial, self-positive, and self-negative reminiscence (Cappeliez and O’Rourke, 2006; O’Rourke et al., 2016). To focus on adaptive usage, this study measures the construct using three scales (Identity, Problem Solving, Death Preparation) within the tripartite model that corresponds to self-positive functions. The internal consistency ranges between *α* = 0.76 and 0.87.

### Prevalence of balancedness, choice of prior

In LDA, a criterion of equal proportions is fundamental because skewed classes would alter the odds and introduce model bias. An even chance provides the least data likelihood for either group and is ideal for determining posterior probability. Updating the posterior from the prior resolves as *X* ~ *U* () ∝ *X* ~ *B* (*p*, *q*).

A series of random draws first levels out balancedness *X* ~ *U* (150, 150) and then divides the resulting observations into subsets of *n* ≈ 240 and *n* ≈ 60 cases. Thus, 0.80 of the observations serve as a training sample, while the remaining 0.20 set up the test cases. Cross-validation builds on test data to evaluate the ability of the model to correctly predict class membership for observations on which the algorithm has not been previously trained (Backhaus et al., 2023; Wirtz, 2021; Rudolf and Buse, 2020).

Aside from median-split, Hierarchical Cluster Analysis (HCA) has also been used in research to capture balancedness (Stolarski et al., 2020; Wiberg et al., 2012; Zhang et al., 2013). Using HCA to classify balancedness generates a skewed prevalence of 189 balanced observations and 133 unbalanced observations, which is *f* (62.6) and *f* (37.4) of sample *N* (302), respectively. Restricted by these initial proportions, subsequent random draws result in small subsets for training *n* (70, 70) and testing *n* (20, 20).

Likewise, further predictors exist, such as HCA 4, median-split, and Wiberg, but they are difficult to interpret as they imply a multinomial differentiation of the criterion. Such a scenario produces multiple equations in LDA, where the number of discriminant functions is given by min [*g* − 1, *k*], with *g* [1; *g*] groups and *k* [1; *k*] predictors (see Table 1).

**Table 1:** Prior probability of balancedness under different operationalizations.

Coefficients	*n*	Balanced	Mean	SD	Median	Trimmed	Min	Max	Range	Skew	Kurtosis	SE
HCA 2^a^	302	Low	1.37	0.48	1.00	1.34	1.00	2.00	1.00	0.52	−1.74	0.03
HCA 4^a^	302	Low	2.19	0.83	2.00	2.19	1.00	4.00	3.00	0.08	−0.81	0.05
Median-split	302	High	2.92	1.40	4.00	3.61	0.00	7.00	7.00	−0.03	−1.66	0.17
Median-split^a^	302	High	1.54	1.22	2.00	1.55	0.00	3.00	4.00	−0.03	−1.59	0.07
Wiberg	302	High	0.66	0.99	0.00	0.46	0.00	3.00	3.00	1.30	0.36	0.06
DBTP	302	Low	3.87	1.40	3.67	3.82	0.19	8.66	8.47	0.50	1.03	0.08
SEM	302	High	0.00	0.70	0.10	0.03	−2.46	1.77	4.22	−0.59	1.25	0.04

a Ordered factor. HCA: Hierarchical Cluster Analysis, 2 or 4 clusters. Semantic of coefficients: HCA, DBTP: [low: balanced, high: unbalanced]; Median-split, Wiberg, SEM: [low: unbalanced, high: balanced]. Median-split: 23 TP = 8 grades of balancedness. Wiberg: compound index, combines ordinal and categorical measures ~ count of TP == 1|*δ* (balanced profile, observed data) ∊ tolerance → 1, either 0; with an adapted definition of tolerance ~ [4.5; 6].

### Operationalizations of BTP

The DBTP coefficient uses formula $\text{DBTP}=\sqrt{\sum {\left(\text{observed}-\text{optimal}\right)}^{2}}$ . However, the scale means from the three time perspectives are used as observed data, with the respective maximum (= 6) specifying the optimal value. The index quantifies a metric measure of balancedness, hence BTP (Stolarski et al., 2011; Vowinckel et al., 2017).

The authors of the present study would also like to propose further operationalization based on a structural equation model. This coefficient relates to a second-order latent variable, estimated by first-order latent constructs of time perspective and life satisfaction, specifically three TP scales and the SWLS measure (SEM; Supplementary materials). Both coefficients, DBTP and SEM, are compared against each other for their ability to classify and predict cases regarding balancedness.

### Statistical analyses

#### Data preparation

Feature engineering ensured the adequacy of predictors in the resulting model. In classification tasks, prior information is fundamental to model accuracy. Thus, attention has been given to how to preset the prevalence of balancedness in the sample. Moreover, feature selection is directed by inferential criteria and the prerequisites of the analysis, as detailed below.

#### Analytical models

The concern of LDA is twofold: first, to classify observations, and second, to predict either a binary or multinomial outcome. LDA expects that the multivariate feature variables are Gaussian. Depending on the respective purpose, the analytical model either classifies cases or predicts the posterior probability for a categorical criterion. A Linear Discriminant Function (LDF) is denoted by *B_j_X*_i_ → *Y*_ig_, where *Y*_ig_ maps to individual discriminant values; *j* [1; *k*] predictors, *i* [1; *n*] observations, *g* [1, *g*] groups. The statistical model is capable of operating complex classifications.

LDA resembles principal components analysis (PCA) in some aspects, as it decomposes multivariate variation and recursively maximizes eigenvalues in vectors described by linear equations. Unlike PCA, where covariation is an emergent property in the data, LDA preserves group membership and integrates the classifier into the model. Thus, the solution vectors also capture psychometric properties (Dunn and Everitt, 2004, pp. 129–198; Rudolf and Buse, 2020, p. 182; Wirtz, 2022b).

#### Inferential decision criteria, exploratory analyses

The use case of discriminant analysis is exploratory for most of the hypotheses, assuming an error level of *α* 0.05 and a Power of 0.80. Rank correlations illustrate the bivariate associations between the feature variables. Their effect size is rated according to individual differences guidelines: low, |*r*| ≤ 0.1; moderate, |*r*| ≤ 0.2; strong, |*r*| ≥ 0.3 (Cohen, 1988, 1992; Gignac and Szodorai, 2016; Leonhart, 2022).

Statistical inference is drawn by: *χ*^2^ to infer the model’s ability to correctly classify and predict observations, *F*-Test and Wilks Lambda to evaluate the eigenvalues of the model and to assess the significance of the discriminant coefficients, and *z*-test to infer the significance of logistic estimates (*Yig*, group membership). A cross-validation procedure segments the sample into a training and a testing subset of *p* (0.80, 0.20). The explained model variance is maximized; lower values on the Akaike Information Criterion (AIC) and Bayesian Information Criterion (BIC) indicate a better fit.

#### Prerequisites of the analytical models

LDA maximizes the explained model variation by a metric discriminant value, which emerges as the eigenvalue from LDF. The centered estimate of this metric specifies the cutoff by which cases are classified. Values greater than (or less than) zero of the categorical criterion are allocated into distinct and homogeneous groups. Notably, multiple LDFs apply in the case of a multinomial criterion. To ensure estimates of good quality, the feature variables must adhere to the following requirements. However, for models with *k* ≤ 5 predictors and *n* ≥ 20 per group, LDA proves to be robust (Backhaus et al., 2023, p. 258; Rudolf and Buse, 2020, p. 181).

- Normally distributed feature variables
- Uniform prevalence of balancedness
- Equal covariance matrices between the groups
- No influential cases in the predictors
- No multicollinearity among the feature variables

#### Multivariate normality

Following data transformation (bestNormalize Package R; D’Agostino and Belanger, 1990; Peterson, 2021; Peterson and Cavanaugh, 2020), measures were normally distributed (past and future TP, SWB, SWLS, SCSKD, PHQ15 Somatoform, PPQ, RFS, SBI, DBTP, SEM, and age). Exclusion of 14 influential cases established on all predictors, including past TP as well (past: *W* = 0.9904, *p* = 0.0617; present: *W* = 0.9935, *p* = 0.264; future: *W* = 0.9941, *p* = 0.347; SWB: *W* = 0.9974, *p* = 0.9316; SWLS: *W* = 0.9951, *p* = 0.5104; DBTP: *W* = 0.9907, *p* = 0.0712; SEM: *W* = 0.9911, *p* = 0.0888; age: *W* = 0.9983, *p* = 0.9951).

A caveat remains for the health measures (PHQ4, PHQ15, ADNM), most likely due to psychometric shortcomings of these scales, for which a limited number of response alternatives suggests ordinal scaling. All of them fail to approximate normality.

#### Uniform prevalence

Applying a median-split procedure establishes an even prevalence among both subsets of unbalanced and balanced observations. Sampling continued to assign cases into a training set (*p =* 0.8) and a testing set (*p* = 0.2), for which equal odds of balancedness prevailed.

#### Homogeneous covariance matrices

This presumption is evaluated by *Box’s M*-test. A non-significant statistic [*χ*^2^ (3) = 2.098, *p* = 0.552] implies homogeneous variances for balanced and unbalanced observations.

#### Influential cases

Cook’s *D* identified 14 influential cases, using either the DBTP or SEM metric; exclusion established normality on all predictors. This finding supports construct validity, since both coefficients are selective for balancedness across all TPs. *Note*: Elimination preserved proportions because outliers were evenly distributed for BTP, thus *X* ~ *U* (143, 143). Observations were randomly assigned to a training data set *n* (114, 117) and a testing data set *n* (30, 29).

#### Multicollinearity

The relative importance of predictors is inferred by the sequence in which the feature variables become relevant, i.e., a stepwise entry into the analytical model. For this purpose, the linear (in-)dependency of predictors is essential to obtain unbiased and stable estimates. Hence, variance inflation (VIF) and tolerance (1/VIF) are indicative. Normalizing transformation reduces collinearity by centering the data and applying unique scaling, suggesting that robustness against redundancy and suppressing effects has been established. As expected, DBTP and SEM are mutually multicollinear; including TP in a model rules out both of them. SWB and SWLS may be analyzed separately, even in the presence of TP. Complying with these restrictions, any model may be estimated as VIF ranges from [1.147; 2.904] (Supplementary materials).

## Results

### Bivariate association among predictors

**Figure 1:**
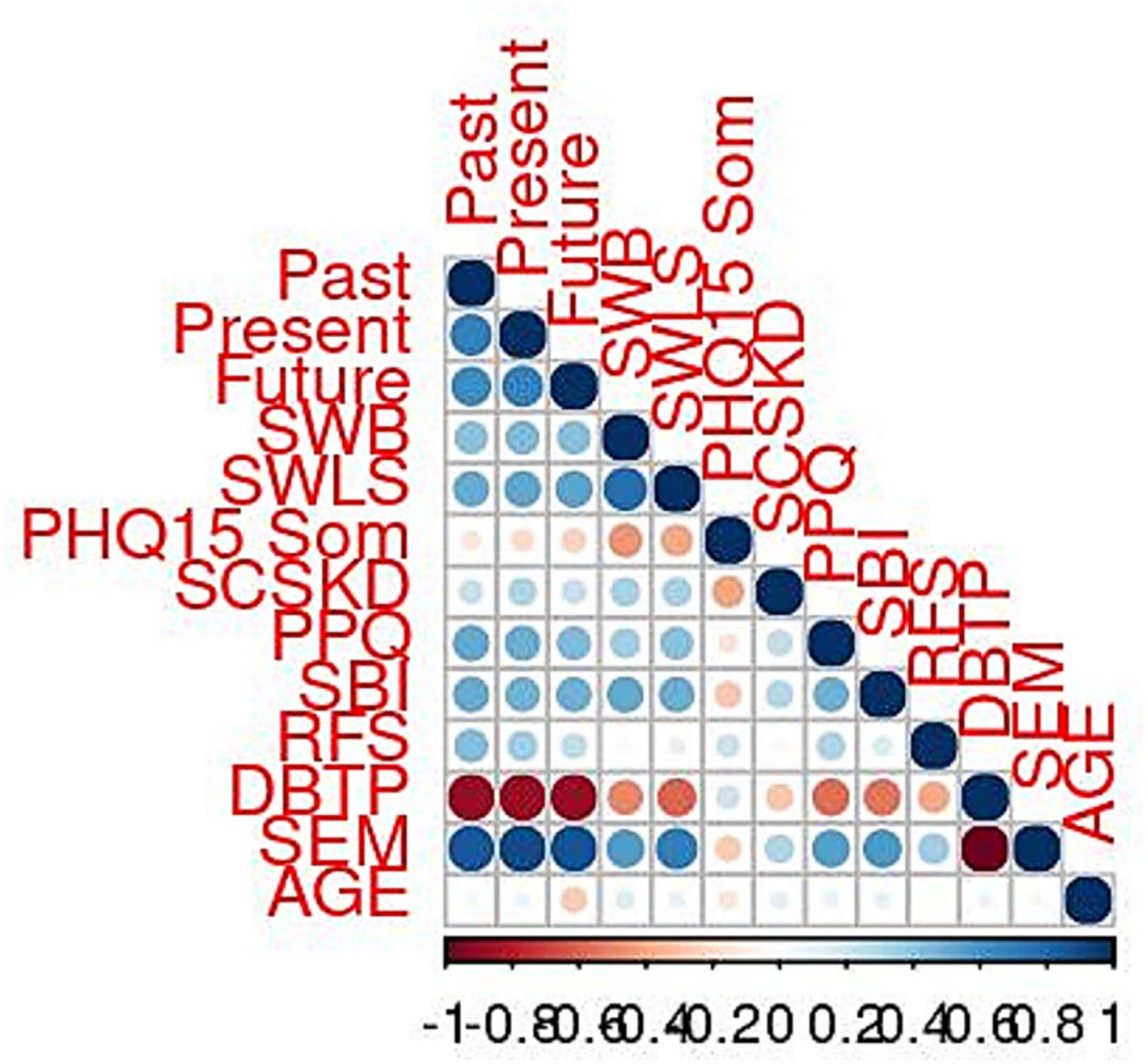
Bivariate associations among feature variables.

**Figure 2:**
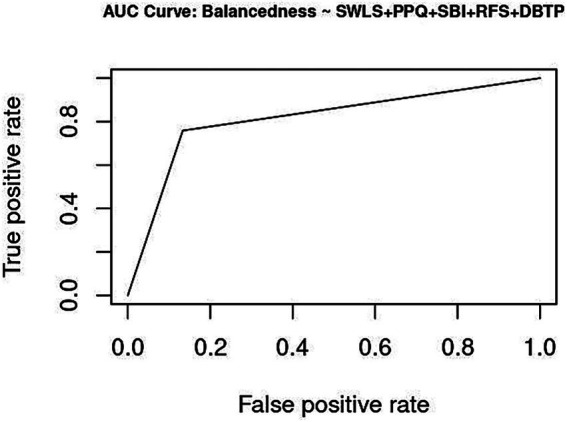
Accuracy: Correct Model Predictions using DBTP.

**Figure 3:**
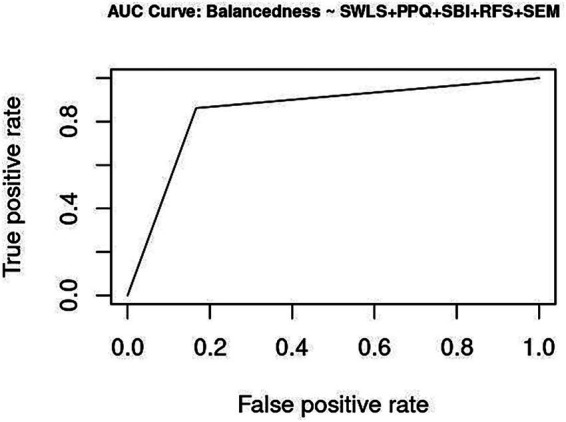
Accuracy: Correct Model Predictions using SEM.

To start with an exploratory overview of the interrelatedness among the feature variables (Figure 1), measures of temporal framing are broadly associated with indicators of well-being and life quality. They also share variance with further covariates of balancedness, such as executive functioning, positivity, and savoring, while health problems covary negatively. Measuring three time perspectives (mBTPS) and the usage of compound indices of balancedness, DBTP and SEM, provide the overall highest covariation. Their association is in the opposite direction; both DBTP and SEM are mutually reverse-signed, due to their respective semantics, which capture distance for the former and proximity for the latter. Age negatively correlates with future TP but shows no relevant covariation with either of the other measures. Contemporary research has verified that sex is not predictive of balancedness (Stolarski et al., 2020), so it is not considered here (see Table 2, Figures 2, 3).

**Table 2:** Bivariate rank correlations.

	Past	Present	Future	SWB	SWLS	PHQ15	SCSKD	PPQ	SBI	RFS	DBTP	SEM	AGE
Past	1												
Present	0.65^***^	1											
Future	0.57^***^	0.65^***^	1										
SWB	0.36^***^	0.43^***^	0.38^***^	1									
SWLS	0.42^***^	0.46^***^	0.44^***^	0.75^***^	1								
PHQ15	−0.11	−0.19^*^	−0.19^*^	−0.42^***^	−0.38^***^	1							
SCSKD	0.18^*^	0.29^***^	0.23^***^	0.33^***^	0.32^***^	−0.39^***^	1						
PPQ	0.50^***^	0.48^***^	0.40^***^	0.35^***^	0.41^***^	−0.14	0.23^**^	1					
SBI	0.44^***^	0.40^***^	0.43^***^	0.46^***^	0.44^***^	−0.25^***^	0.27^***^	0.47^***^	1				
RFS	0.39^***^	0.30^***^	0.21^**^	0.02	0.05	0.21^**^	−0.06	0.25^***^	0.09	1			
DBTP	−0.85^***^	−0.84^***^	−0.85^***^	−0.44^***^	−0.50^***^	0.20^**^	−0.27^***^	−0.53^***^	−0.46^***^	−0.32^***^	1		
SEM	0.81^***^	0.88^***^	0.84^***^	0.53^***^	0.62^***^	−0.24^***^	0.32^***^	0.55^***^	0.51^***^	0.30^***^	−0.98^***^	1	
AGE	0.03	0.06	−0.25^***^	0.14	0.09	−0.12	0.11	0.09	0.10	0.02	0.07	−0.04	1

^*^p < 0.05, ^**^p < 0.01, ^***^p < 0.001 (effect sizes: Gignac and Szodorai, 2016).

Rank correlations were conducted to adequately report covariation and to gain consistent estimates as the measures are of both metric and categorical scale levels (Table 3). Overall, significant coefficients are of moderate to strong effect size. Age is negatively correlated with future TP (*r* = −0.25, *p* ≤ 0.001) but is not associated with either of the other measures.

**Table 3:** Multivariate statistics of the LDF equation.

	*ᴦ*	Wilks *λ*, *χ*^2^(df)	*F*-statistic	AIC	*ᴦ*	Wilks *λ*, *χ*^2^ (df)	*F*-statistic	AIC
Median-split	DBTP				SEM			
Analytical model DBTP	0.6015	*λ* = 0.3985	*F*(5, 225) = 68.22	102.07	NA	NA	NA	NA
		*χ*^2^ (5) = 208.39						
			*p* < 0.0001					
		*p* < 0.0001						
Analytical model SEM	NA	NA	NA	NA	0.6234	*λ* = 0.3766	*F*(5, 225) = 74.82	87.24
						*χ*^2^ (5) = 221.19		
							*p* < 0.0001	
						*p* < 0.0001		

Prevalence by Median-split, for some models compared to binary clustering (HCA2). Gamma: standardized eigenvalue of the LDF, expresses explained variance.

Except for age and the health measure (PHQ15), temporal framing, as assessed by the three TP scales, significantly correlates with all constructs at a moderate level. It has the strongest effects for DBTP (past: *r* = −0.85; present: *r* = −0.84; future: *r* = −0.85, all: *p* ≤ 0.001) and SEM (past: *r* = 0.81; present: *r* = 0.88; future: *r* = 0.84, all: *p* ≤ 0.001), followed by Prioritizing Positivity (past: *r* = 0.50; present: *r* = 0.48; future: *r* = 0.40, all: *p* ≤ 0.001), Savoring Beliefs (past: *r* = 0.44; present: *r* = 0.40; future: *r* = 0.43, all: *p* ≤ 0.001), and both well-being measures, with SWLS showing marginally larger effects (past: *r* = 0.42; present: *r* = 0.46; future: *r* = 0.44, all: *p* ≤ 0.001) than Subjective Well-being (past: *r* = 0.36; present: *r* = 0.43; future: *r* = 0.38, all: *p* ≤ 0.001). Replacing the originating TP scales with either DBTP or SEM yields consistent correlations of equal size with these measures.

RFS does not covary with well-being (assessed by SWLS, SWB), nor with executive functioning (SCSKD) or savoring beliefs (SBI). Unlike TP scales, reminiscence positively correlates at an average moderate level (past: *r* = 0.39, *p* ≤ 0.001; present: *r* = 0.30, *p* ≤ 0.001; future: *r* = 0.21, *p* < 0.05), as well as with PHQ15 (*r* = 0.21, *p* ≤ 0.05) and prioritizing positivity (*r* = 0.25, *p* ≤ 0.001).

### Analytical models

LDA aims to assess how selective the three TPs are towards balance and to compare the two compound coefficients of DBTP and SEM. Data likelihood provides insight into the explanatory properties of the classifiers as expressed in model fit. Evidence is collected for a set of feature variables and will be cross-validated on test data to assess the predictive ability of the model. Parsimony and group size adhere to formal constraints of the statistical model while ensuring that an even prevalence of balance is maintained between the subsets. Median-split is applied to preset the prior, *X* ~ *U* (0.494, 0.506).

Comparing three versus two TPs yields a standardized eigenvalue of 0.5040 for BTPS (*F* (2, 228) = 116.38, *p* <. 001; Wilks *λ* = 0.4959, *χ*^2^ (2) = 159.92, *p* < 0.001; AIC 130.86) and 0.5751 for mBTPS [*F* (3, 227) = 102.85, *p* < 0.001; Wilks *λ* = 0.4249, *χ*^2^ (3) = 194.72, *p* < 0.001; AIC 73.35]. Accordingly, the explained model variance incrementally increases by 0.08 for mBTPS over BTPS. Therefore, data likelihood is higher in a model of three TPs compared to two TPs.

Replacing the originating TP scales in mBTPS with a compound indicator of balancedness results in a close surplus of an additional 0.02 explained model variance. The usage of DBTP has an eigenvalue of 0.5952 [*F* (1, 229) = 338.10, *p* < 0.001; Wilks *λ* = 0.4048, *χ*^2^ (1) = 206.65, *p* < 0.001; AIC 97.56], and SEM converges at 0.5845 [*F* (1, 229) = 323.60, *p* < 0.001; Wilks *λ* = 0.4155, *χ*^2^ (1) = 200.69, *p* < 0.0001; AIC 107.90].

Model fit improves further when prevalence is initially clustered by HCA: up to 0.6397 for the DBTP coefficient [*F* (1, 134) = 239.64, *p* < 0.0001; Wilks *λ* = 0.3603, *χ*^2^ (1) = 134.24, *p* < 0.0001; AIC 42.45], and 0.6131 for the SEM coefficient [*F* (1, 134) = 252.90, *p* < 0.001; Wilks *λ* = 0.3869, *χ*^2^ (1) = 124.87, *p* < 0.0001; AIC 36.35]. The downside of this approach is that it yields a distinctly skewed prior of *n* = 189 balanced observations. Aiming for even prevalence will also result in small subsets. In addition, this type of model carries the risk of being overfitted.

Therefore, prevalence is still allocated by median-split. However, relying on a single feature variable for modeling is probably too sparse, suggesting that the LDF will benefit from additional predictors to enhance its selective quality.

### Comprehensive evaluation of intermediary results

Up to this point, the goal of feature engineering has been to select predictors that are likely suitable for quantifying the degree to which DBTP or SEM usage outperforms time perspective scales, BTPS and mBTPS, and to evaluate additional covariates of balancedness. Preliminary findings suggest that estimates of both indicators, DBTP and SEM, yield similar data likelihood (Supplementary materials). All appropriate measures will be entered into a comprehensive final model that evaluates selectivity and predictive accuracy. Hence, the analyses will first be conducted with a DBTP metric and then compared to a structurally equivalent model that employs SEM.

Training the models to fit the data provides evidence of how well the measures can describe the observations in terms of balancedness. An eligible classifier will be able to distinctly separate cases and allocate group membership. To be entered into the final analytical model, a measure must be both selective and predictive. In LDA, multivariate analyses of variance (MANOVA) and Wilks Lambda assess model fit: univariate analyses of variance (ANOVA) evaluate the significance of discriminant coefficients, and logistic regression estimates the predictive power of a predictor. Failing to meet either of these criteria excludes a feature variable from subsequent analyses. Accordingly, age is not sensitive enough to consistently capture states of balancedness across all time perspectives [*F* (1, 229) = 0.029, *ns; b* = −0.*023, ns*], nor do PHQ15 [*F* (1, 229) = 3.020, *ns; b* = −0.240, ns], SWB [*F* (1, 229) = 19.66, *p* < 0.0001; *b* = 0.01453, *ns*], and SCSKD [*F* (1, 229) = 3.075, *ns; b* = −0.057, *p* = 0.045] perform adequately (Supplementary materials). The remaining set of measures specifies the target model. As mBTPS has already been shown to outperform BTPS in terms of model fit, the coefficients of DBTP and SEM are operationalized across all three TP. Hence, two models are being compared:

$$\begin{array}{l} \text{Balancedness}\sim \text{SWLS}+\mathrm{PPQ}+\mathrm{SBI}+\mathrm{RFS}\\ +\text{DBTP and Balancedness}\sim \text{SWLS}\\ +\mathrm{PPQ}+\mathrm{SBI}+\mathrm{RFS}+\mathrm{SEM}. \end{array}$$

Overall, both models are capable of classifying cases and separating unbalanced from balanced observations. Multivariate model tests report a standardized eigenvalue of 0.6015 for DBTP [*F* (5, 225) = 68.22, *p* < 0.0001; Wilks *λ* = 0.3985, χ^2^ (5) = 208.39, *p* < 0.0001; AIC 102.07], while SEM yields a share of 0.6234 explained variance [*F* (5, 225) = 74.82, *p* < 0.001; Wilks *λ* = 0.3766, *χ*^2^ (5) = 221.19, *p* < 0.001; AIC 87.42] (see Table 4).

**Table 4:** Inferential statistics of the discriminant coefficients.

	Centroids balanced [0; 1]	LDF coefficients	*F*-statistic *F* (1, 229)	Log. coefficients beta, *p*
DBTP				
SWLS	−0.3879; 0.3617	−1.6843	35.65, *p* < 0.0001	−0.2741, *p = 0*.523
PPQ	−0.5125; 0.4412	0.01907	65.69, *p* < 0.0001	0.0281, *p = 0*.946
SBI	−0.4358; 0.3411	−0.04801	38.85, *p* < 0.0001	0.0392, *p = 0*.921
RFS	−0.2866; 0.3067	−0.07572	21.58, *p* < 0.0001	0.5317, *p = 0*.108
DBTP	0.8114; −0.8119	1.62785	338.14, *p* < 0.0001	−6.0971, *p* < 0.0001
SEM				
SWLS	−0.3820; 0.3618	−0.5107	34.87, *p* <. 0001	−2.6648, *p* = 0.0001
PPQ	−0.5137; 0.4412	0.01129	65.89, *p* <. 0001	0.0392, *p* = 0.927
SBI	−0.4416; 0.3411	−0.1036	39.30, *p* <. 0001	−0.0856, *p* = 0.839
RFS	−0.2952; 0.3067	−0.1024	22.45, *p* <. 0001	0.4969, *p = 0*.166
SEM	−0.8061; 0.8051	1.91035	323.56, *p* < 0.0001	9.1032, *p* < 0.0001

Though initial analyses suggest an overall fit of PPQ, SBI, and RFS, the analytical model does not fully support this view. While these feature variables perform well in selectively classifying observations for balancedness, they account for only small shares of overall model variance. Correspondingly, discriminant coefficients (Table 5) express relevance in the resulting discriminant value of the LDF (centered at zero). Higher values indicate precedence. Ordering predictors by precedence metric indicators holds distinct relevance in both models, followed by well-being. In general, measures may contribute to overall model fit (significant *F*-statistic) but are not as effective in prediction (non-significant logistic estimate). Results indicate that DBTP [−1.628; *F* (1, 229) = 338.14, *p* < 0.001; *b* = −6.097, *p* < 0.001] and SEM [1.910; *F* (1, 229) = 323.56, *p* < 0.001; *b* = 9.103, *p* < 0.001] are relevant classifiers and significant predictors of balancedness; the same applies to SWLS. More specifically, SWLS is both selective and predictive, aside from SEM [−0.5107; *F* (1, 229) = 34.87, *p* < 0.001; *b* = −2.665, *p* < 0.001], but is only selective apart from DBTP [−0.1684; *F* (1, 229) = 35.65, *p* < 0.001; *b* = −0.274, *ns*].

**Table 5:** Cross-validated accuracy of predictions.

Predicted observed	DBTP		SEM		Margin	npv	ppv
	0	1	0	1			
0	103	11	102	12	114 (49.40)	DBTP; SEM 0.904; 0.895	
1	8	109	6	111	117 (50.60)		DBTP; SEM 0.932; 0.949
Margin	110 (47.60)	121 (52.40)	108 (46.80)	123 (53.20)	231 (100.0)		
Sensitivity	0.936		0.944				
Specificity		0.901		0.902			

Balanced [0; 1]; positive predictive value (ppv), negative predictive value (npv).

Univariate ANOVA contrasts indicate that average ratings on SWB [unbalanced: −0.2981 (63.56); balanced: 0.2610 (75.92)] differ between levels of balancedness [*F* (1, 229) = 19.86, *p* < 0.001]. This effect is not moderated by age (*b* = 0.085, *ns*), but interacts with temporal framing (*b* = 0.624, *p* < 0.001). The level of experienced life quality predicts balancedness (*b* = 0.822, *p* < 0.0001).

Both models are evaluated for their ability to correctly classify and predict balancedness. Cross-validating the trained estimates with a testing subset provides evidence of model accuracy. Selectivity can further be decomposed into sensitivity and specificity. A trade-off between false discoveries (Type I error) and false omissions (Type II error) will comparatively evaluate the performance of DBTP and SEM for their respective positive and negative predictive values. Predictive accuracy is strongly dependent on prevalence (Cohen et al., 2016; Murad et al., 2023).

Five-fold cross-validation was conducted on a test sample, for which forecasts of balancedness were parameterized by the estimates from the training data. Both indicators perform very well: DBTP estimates have 0.918 correct predictions [*χ*^2^ (1) = 158, *p* < 0.0001] and a precision of 0.922 for SEM [*χ*^2^ (1) = 161.63, *p* < 0.0001].

Youden Index specifies cutoff values for which the Area Under the Curve (AUC) is maximized [∫sensitivity × (1 − specificity) → max!]. DBTP converges at 0.8363, and SEM at 0.8548. For a binary criterion, the distance between the Youden estimate and 1 quantifies the range in which a categorical criterion misclassifies. Overall, both models demonstrate very good to excellent performance within the upper percentile of precise estimations. Specifically, SEM shows slightly better performance in predicting the presence of balancedness, whereas DBTP is marginally more accurate in indicating its absence (Supplementary materials).

## Discussion

The findings largely support the idea that balancedness in temporal framing is adaptive and influential in well-being and associated covariates, such as positivity and indicators of selective goal attention. Reminiscence is relevant across all three temporal directions but does not correlate with either well-being measure; rather, it is associated with both the health measure and prioritizing positivity. In accordance with the tripartite model of reminiscence functions, the results provide evidence that the impact of reminiscence on life quality and health is seemingly indirect, relating to selective goal attention (McLean et al., 2020; O’Rourke et al., 2010; O’Rourke et al., 2016). Moreover, corresponding to established evidence on balancedness as a broadly acknowledged covariate of well-being, aggregated indicators such as DBTP predict balancedness better than scale-based operationalizations (Stolarski et al., 2020). Hence, replacing TP scales with compound indices of time perspective improves overall fit in explained model variance, but at the scale level, covariation remains of equal size regarding both DBTP and SEM and the remaining measures.

The study compares feature variables for their capacity to accurately capture time perspectives and balancedness in temporal framing. Initially, the choice of feature variables is conceptually founded on covariates of balancedness (Stolarski et al., 2020), while subsequent quality selection is data-driven and modeled by LDA. The respective analytical models address open questions in research: (a) the construct dimensionality of balancedness in temporal framing and whether two or three time perspectives are required to assess psychological processes related to well-being and life quality; (b) the adequacy of metrics, which has been broadly discussed for categorical indices (median-split), ordinal measures (Wiberg coefficient), and metric indicators (DBTP) of balancedness (Webster, 2011; Wiberg et al., 2012; Zhang et al., 2013; Zimbardo et al., 2019). The SEM coefficient has previously been introduced in our research project and is now compared for incremental performance against DBTP.

It is presumed that indicators on a metric scale generally outperform categorical or ordinal measures, arguing that different approaches must be evaluated under the constraint of prevalence in order to gain precise estimates (Döring, 2022; Kuhn and Johnson, 2020). Sensitivity and specificity, or a compound measure of both (AUC), are used to quantify model accuracy in predicting balancedness (Goldhammer and Hartig, 2020, pp. 179–183). A five-fold cross-validation is applied to infer the accuracy of model predictions.

Comparing three versus two time perspectives, mBTPS exceeds BTPS with a surplus of 0.08 explained variance (Vowinckel et al., 2017). Substituting TP scales with either DBTP or SEM slightly improves model fit. However, the initial assumption that balancedness is established by executive functioning is not supported by the findings, as SCSKD is ruled out during the feature engineering process and thus is not considered in the analytical model. While cognitive ability is generally adaptive, reserve capacity must be controlled for in order to assess the impact of self-regulation on balancedness (Chuderski et al., 2012; Stolarski et al., 2020). Age negatively covaries with future TP, but neither varies across the 6 age groups [18; 71+] nor systematically impacts the remaining measures.

In accordance with state-of-the-art knowledge, balancedness in temporal framing is associated with well-being and life quality (Burzynska and Stolarski, 2020; Przepiorka and Sobol-Kwapinska, 2021). In both analytical models, well-being yields high predictive precedence in overall model fit. However, whereas SWLS is selective and predictive of balancedness aside from SEM, it is only selective apart from DBTP. The mean experience of subjective well-being differs with regard to balancedness. In balanced observations, SWB ranges in the upper value quartile and is lower otherwise. The corresponding threshold is proposed as a cutoff with clinical implications (Cummins et al., 2019).

Although all feature variables significantly contribute to model fit, balancedness is particularly well-captured by well-being aside from either DBTP or SEM. The examination of logistic estimates suggests the redundancy of PPQ, SBI, and RFS, so these measures can be omitted in a parsimonious model. Notwithstanding that the experience of well-being is fundamental in positive psychology, the construct clearly encompasses more than just happiness (Webster and Vowinckel, 2021), as results underscore that positivity and balancedness are mutually distinct concepts. Therefore, a parsimonious model is sufficiently specified by well-being in conjunction with either DBTP or SEM. A balanced TP can be captured by a metric index at best, which is in accordance with current evidence (Zhang et al., 2013).

The results indicate that the SEM coefficient is slightly more accurate in detecting the presence of balancedness, as opposed to DBTP, which captures its absence marginally better. The two coefficients differ with regard to their semantics; the former is founded on proximity, while the latter indicates distance.

## Limitations

The results may be specific to a scientific sample, primarily consisting of panelists. However, the age range and socioeconomic status of the participants are broadly diverse, although the evidence is limited to Western European adults. In addition, self-report measures are prone to biases of motivational or informational origin. Nonetheless, no systematic effect is expected as benefits are granted independently from the answers, and quality items ensure accuracy in the responses. A conclusion about causality cannot be drawn, as a cross-sectional design does not allow for the differentiation of predictor and criterion for cause and effect. While this methodological consideration is generally applicable, external cross-validation does indicate excellent model accuracy. The magnitude of selectivity, specificity, and predictive values is excellent. Covariation suggests that balancedness relates to processes of selective goal attention. However, the recursive nature of biographical memory across experiences in the lifespan leaves open the question of whether balancedness or well-being emerges bottom–up or top–down (Cunningham et al., 2015). More specifically, either living conditions improve well-being, or traits foster adaptive habits; both directions accumulate resources and contribute to psychological relevance (Boniwell et al., 2010; Roglic, 2016; Stolarski et al., 2014). This suggests that the effect on well-being is indirect and should be studied using mediation analyses. In the case of reciprocal interdependence, cross-lagged models and experimental designs provide insight into causality and moderation (Burzynska and Stolarski, 2020). Finally, the psychometric properties remain an open issue regarding whether balancedness is a state or a trait (Stolarski et al., 2018, 2020).

## Conclusion

Evidence suggests the psychological relevance of three time perspectives: past, present, and future TP. A parsimonious model preferably uses a compound measure, DBTP or SEM, to evaluate balancedness. Hence, mBTPS outperforms BTPS. In terms of explained model variance, the latter two indices incrementally improve parameter estimation, as opposed to using TP scales. As both indices are mutually multicollinear, a model may contain only one of them. However, complementary indication is advised as DBTP has a slightly greater predictive value for the absence of balancedness, whereas the SEM captures its presence marginally better. Both concepts, well-being and life quality, provide high-quality estimates of balancedness. In the analytical models, this is DBTP or SEM aside from SWLS.

Additional findings support that the upper quartile in average ratings on subjective well-being has clinical relevance. Age does not moderate this effect; it rather depends on the time perspective used in temporal framing.

Prior information should be taken into consideration first, rather than focusing on scale levels in measures and indicators. Cross-validating a model is strongly recommended to evaluate model accuracy. In this study, the percentage of true assignments falls within the upper percentile of precise estimates for both classification and prediction.

## Data Availability

The study and its design had been preregistered. The datasets presented in this study can be accessed in online repositories. The names of the repository/repositories and accession number(s) can be found at: https://osf.io/9p2yc/?view_only=b30358166b6a4e33beace6a47109c90e.

## Footnotes

1
https://mingle.respondi.de
