# Supplementary material for: The three-dimensional structure of temporal framing in European adults: balancedness in past, future, and present time perspective
Source: Front Psychol. 2026 May 11;17:1722605. doi: 10.3389/fpsyg.2026.1722605 (PMC13199323; doi:10.3389/fpsyg.2026.1722605)
Supplement: Supplementary file 1 [file Supplementary_file_1.docx]

**Title**

**Balanced Time Perspective and Covariates of Well-being**

**Supplementary Materials**

*Table S1: Equal Distribution of Age and Sex*

| Age Group |  |  | Sex | |  |  |
| --- | --- | --- | --- | --- | --- | --- |
|  | male | | female | | divers | |
|  | *n* | % | *n* | % | *n* | % |
| 18-30 | 13 | 26.0 | 36 | 72.0 | 1 | 2.0 |
| 31-40 | 21 | 42.0 | 29 | 58.0 | 0 | 0.0 |
| 41-50 | 25 | 50.0 | 25 | 50.0 | 0 | 0.0 |
| 51-60 | 31 | 62.0 | 19 | 38.0 | 0 | 0.0 |
| 61-70 | 32 | 64.0 | 18 | 36.0 | 0 | 0.0 |
| 71+ | 28 | 53.8 | 24 | 46.2 | 0 | 0.0 |
|  |  |  |  |  |  |  |
| Σ | 150 | 49.7 | 151 | 50.0 | 1 | 0.3 |

*Note:* Age × Sex [ 2(5) = 19.18, *p* = .002 ]

*Table S2: Distributional Parameters of Normalized Feature Variables*

| Feature variables | n | *X*~*N*( ) | mean | sd | median | trimmed | min | max | range | skew | kurtosis | se |
| --- | --- | --- | --- | --- | --- | --- | --- | --- | --- | --- | --- | --- |
| Past | 302 | yes | 0.00 | 1.00 | 0.08 | 0.04 | -2.95 | 2.42 | 5.37 | -0.46 | 0.51 | 0.06 |
| Present | 302 | yes | 0.00 | 1.00 | 0.09 | -0.01 | -2.71 | 2.96 | 5.68 | -0.02 | 0.36 | 0.06 |
| Future | 302 | yes | 0.00 | 1.00 | 0.02 | 0.00 | -2.72 | 2.94 | 5.65 | -0.01 | -0.12 | 0.06 |
| SWB | 302 | yes | 0.00 | 0.99 | 0.05 | 0.00 | -2.93 | 2.57 | 5.50 | -0.02 | -0.12 | 0.06 |
| SWLS | 302 | yes | 0.00 | 1.00 | -0.03 | 0.00 | -2.58 | 2.58 | 5.16 | -0.01 | -0.17 | 0.06 |
| PHQ15 Somatoform | 302 | yes | 0.01 | 0.96 | -0.03 | -0.01 | -1.57 | 2.94 | 4.51 | 0.19 | -0.41 | 0.06 |
| SCSKD, onedimensional | 302 | yes | 0.00 | 1.00 | 0.02 | 0.00 | -2.94 | 2.94 | 5.87 | 0.00 | -0.09 | 0.06 |
| PPQ, onedimensional | 302 | yes | 0.00 | 1.00 | 0.04 | 0.00 | -2.94 | 2.72 | 5.65 | -0.01 | -0.11 | 0.06 |
| RFS, general factor | 302 | yes | 0.00 | 1.00 | -0.02 | 0.00 | -2.58 | 2.94 | 5.52 | 0.01 | -0.13 | 0.06 |
| SBI, general factor | 302 | yes | 0.00 | 1.00 | -0.01 | 0.00 | -2.94 | 2.94 | 5.87 | 0.00 | -0.09 | 0.06 |
| DBTP | 302 | no | 0.00 | 1.00 | 0.00 | 0.00 | -2.94 | 2.94 | 5.87 | 0.00 | -0.09 | 0.06 |
| SEM | 302 | yes | 0.00 | 1.00 | 0.00 | 0.00 | -2.94 | 2.94 | 5.87 | 0.00 | -0.09 | 0.06 |
| AGE | 302 | yes | 0.00 | 1.00 | 0.00 | 0.00 | -2.94 | 2.94 | 5.87 | -0.01 | -0.13 | 0.06 |

*Note*: *X*~*N*( ). See supplementary materials for data before normalizing transformation

*Table S3: Distributional Parameters, Raw Data*

| Feature variables | n | *X*~*N*( ) | mean | sd | median | trimmed | min | max | range | skew | kurtosis | se |
| --- | --- | --- | --- | --- | --- | --- | --- | --- | --- | --- | --- | --- |
| Past (1) | 302 | yes | 3.71 | 0.92 | 3.79 | 3.75 | 1.00 | 5.93 | 4.93 | -0.46 | 0.51 | 0.05 |
| Present | 302 | yes | 3.87 | 0.84 | 4.00 | 3.91 | 1.00 | 6.00 | 5.00 | -0.53 | 0.95 | 0.05 |
| Future | 302 | yes | 3.85 | 1.01 | 4.00 | 3.91 | 1.00 | 6.00 | 5.00 | -0.64 | 0.48 | 0.06 |
| SWB01 (2) | 302 | yes | 69.78 | 19.76 | 75.00 | 72.03 | 0.00 | 100.00 | 100.00 | -1.05 | 0.97 | 1.14 |
| SWB01 (3) | 294 | yes | 69.83 | 19.93 | 75.00 | 72.11 | 0.00 | 100.00 | 100.00 | -1.05 | 0.95 | 1.16 |
| SWLS | 302 | yes | 4.42 | 1.22 | 4.40 | 4.48 | 1.00 | 7.00 | 6.00 | -0.40 | -0.05 | 0.07 |
| PHQ4 | 302 | no | 0.78 | 0.69 | 0.75 | 0.69 | 0.00 | 3.00 | 3.00 | 1.00 | 0.86 | 0.04 |
| PHQ4 Anxiety | 302 | no | 0.76 | 0.74 | 0.50 | 0.65 | 0.00 | 3.00 | 3.00 | 0.99 | 0.65 | 0.04 |
| PHQ4 Depression | 302 | no | 0.81 | 0.74 | 1.00 | 0.71 | 0.00 | 3.00 | 3.00 | 0.92 | 0.61 | 0.04 |
| PHQ15 | 302 | no | 0.50 | 0.36 | 0.40 | 0.47 | 0.00 | 1.67 | 1.67 | 0.69 | -0.31 | 0.02 |
| PHQ15 Somatic | 302 | yes | 0.40 | 0.32 | 0.31 | 0.37 | 0.00 | 1.50 | 1.50 | 0.85 | 0.07 | 0.02 |
| PHQ15 Depression | 302 | no | 1.13 | 0.83 | 1.00 | 1.07 | 0.00 | 3.00 | 3.00 | 0.61 | -0.37 | 0.05 |
| ADNM | 162 | no | 3.04 | 0.71 | 3.12 | 3.06 | 1.50 | 5.12 | 3.62 | -0.10 | -0.47 | 0.06 |
| ADNM Preoccupation | 62 | no | 3.34 | 0.59 | 3.50 | 3.39 | 1.75 | 4.25 | 2.50 | -0.71 | -0.32 | 0.05 |
| ADNM Maladaptation | 162 | yes | 2.75 | 1.01 | 2.75 | 2.72 | 1.00 | 6.50 | 5.50 | 0.55 | 0.83 | 0.08 |
| SCSKD | 302 | yes | 4.57 | 0.87 | 4.54 | 4.55 | 0.90 | 2.46 | 6.92 | 4.46 | 0.20 | -0.30 |
| GQ5 | 301 | no | 4.92 | 1.08 | 5.00 | 4.96 | 1.19 | 1.00 | 7.00 | 6.00 | -0.34 | 0.09 |
| PPQ | 302 | yes | 6.05 | 1.38 | 6.17 | 6.12 | 1.46 | 1.00 | 9.00 | 8.00 | -0.54 | 0.46 |
| RFS | 302 | yes | 3.44 | 0.89 | 3.45 | 3.46 | 0.82 | 1.00 | 6.00 | 5.00 | -0.18 | 0.16 |
| RFS Identity | 302 | no | 3.86 | 0.99 | 4.00 | 3.92 | 0.99 | 1.00 | 6.00 | 5.00 | -0.59 | 0.36 |
| RFS Problem Solving | 302 | no | 4.02 | 0.97 | 4.25 | 4.09 | 0.74 | 1.00 | 6.00 | 5.00 | -0.70 | 0.63 |
| RFS Death Preparation | 302 | no | 2.55 | 1.22 | 2.29 | 2.46 | 1.42 | 1.00 | 6.00 | 5.00 | 0.51 | -0.66 |
| SBI | 302 | yes | 4.78 | 0.84 | 4.71 | 4.76 | 0.93 | 2.04 | 6.83 | 4.79 | 0.08 | -0.24 |
| SBI Anticipating | 302 | yes | 4.76 | 0.91 | 4.75 | 4.73 | 1.11 | 2.12 | 6.88 | 4.76 | 0.18 | -0.29 |
| SBI Savoring | 302 | no | 4.70 | 0.99 | 4.62 | 4.69 | 0.93 | 1.88 | 6.88 | 5.00 | 0.10 | -0.32 |
| SBI Reminiscing | 302 | yes | 4.89 | 0.90 | 4.88 | 4.88 | 1.10 | 2.12 | 7.00 | 4.88 | -0.06 | -0.23 |
| DBTP | 302 | no | 3.87 | 1.40 | 3.67 | 3.82 | 0.19 | 8.66 | 8.47 | 0.50 | 1.03 | 0.08 |
| SEM | 302 | yes | 0.00 | 0.70 | 0.10 | 0.03 | -2.46 | 1.77 | 4.22 | -0.59 | 1.25 | 0.04 |
| AGE | 302 | yes | 50.54 | 17.49 | 51.00 | 50.73 | 18.00 | 81.00 | 63.00 | -0.06 | -1.20 | 1.01 |

*Note:* *X*~*N*( ) succeeding normalizing data transformation; (1) past TP: excluding n = 14 outliers; (2) MCAR: *χ^2^* (1) = 0.122, *p* = .727; (3) 8 cases NA

*Table S4: Multicollinearity of Feature Variables*

|  | excluding  DBTP, SEM | | all predictors | | DBTP only | | SEM only | |
| --- | --- | --- | --- | --- | --- | --- | --- | --- |
| Measure/ Scale(s) | vif | tol. | vif | tol. | vif | tol. | vif | tol. |
| Past | 2.480 | 0.403 | 11.331 | 0.088 | 10.890 | 0.092 | 8.497 | 0.118 |
| Present | 2.550 | 0.393 | 13.411 | 0.075 | 6.898 | 0.145 | 13.371 | 0.075 |
| Future | 2.710 | 0.369 | 11.381 | 0.088 | 9.975 | 0.100 | 10.003 | 0.091 |
| SWB | 2.672 | 0.374 | 2.721 | 0.368 | 2.677 | 0.374 | 2.712 | 0.369 |
| SWLS | 2.904 | 0.344 | 5.517 | 0.182 | 2.911 | 0.344 | 4.170 | 0.240 |
| PHQ15 general factor | 1.390 | 0.720 | 1.393 | 0.728 | 1.392 | 0.718 | 1.393 | 0.718 |
| SCSKD | 1.147 | 0.872 | 1.147 | 0.872 | 1.147 | 0.072 | 1.147 | 0.072 |
| PPQ | 1.650 | 0.606 | 1.722 | 0.581 | 1.722 | 0.581 | 1.688 | 0.593 |
| RFS, general factor | 1.490 | 0.673 | 1.525 | 0.656 | 1.523 | 0.656 | 1.497 | 0.668 |
| SBI, general factor | 1.720 | 0.583 | 1.877 | 0.533 | 1.832 | 0.550 | 1.722 | 0.581 |
| DBTP | NA | NA | 83.454 | 0.012 | 44.965 | 0.022 | NA | NA |
| SEM | NA | NA | 128.651 | 0.008 | NA | NA | 69.318 | 0.014 |
| AGE | 1.285 | 0.778 | 1.310 | 0.764 | 1.294 | 0.773 | 1.310 | 0.764 |

*Table S5: Scale Analyses*

|  | Cronbachs Alpha | McDonalds Omega |  | multiple *R*^2^:  factor scores | |
| --- | --- | --- | --- | --- | --- |
| Measure/ Scale(s) |  | general | subscales | general | subscales |
| Past | .95 | .95 |  | .95 |  |
| Present | .91 | .92 |  | .92 |  |
| Future | .96 | .98 |  | .96 |  |
| mBTPS/ BTPS: past; present; future | .97 | .77 | .57; .58; .65 | .78 | .69; .67; .55 |
| SWLS | .91 | .91 |  | .92 |  |
| PHQ4: gen., Anxiety, Depression | .88; .80; .84 | .84 | .70; .75 | .84 | .19; .20 |
| PHQ15: gen., Somatoform, Depression | .83; .80; .75 | .45 | .38; .28 | .48 | .54; .39 |
| ADNM: gen., Preoccupation, Maladaptation | .80; .72; .78 | .62 | .47; .49 | .64 | .42; .44 |
| SCSKD | .82 | .82 |  | .84 |  |
| GQ5 | .82 | .85 |  | .85 |  |
| PPQ | .82 | .82 |  | .84 |  |
| RFS: gen.; Identity, Prbl. Solving, Death Prep. | .89 | .69 | .69; .23; .73 | .82 | .53; .84; .16 |
| SBI: gen., 1st factor, 2nd factor, 3rd factor | .93 | .62 | .33; .65; .36 | .70 | .77; .31; .71 |

*Note*: To assess scales consistency, Cronbachs α and Mc Donalds ⍵ were performed. While the former index points towards an overall good up to excellent reliability, so does the latter picture more details. To begin with, exploratory factor analyses contradict a simple structure for some of the measures, i.e. RFS and SBI for which subscales have crossloadings. Hence, to infer meaningful factors was either impossible or additional inconsistencies occurred, for instance a one-item factor, where the originating scale is constructed by four items loading on it (RFS, subscale Problem Solving). Consequently, a general factor model of these scales has bad fit (RFS: SRMR .17, RMSEA .199 90% CI [.185;.214], BIC 317.83; SBI: SRMR .17, RMSEA .138 90% CI [.132;.144], BIC 258.61). PHQ15: only the Somatoform subscale has sufficient reliability, but not a general factor model.

*Table S6: Inferential Statistics of the Discriminant Coefficients, DBTP modeled*

|  | Centroids  balanced [0; 1] | LDF coefficients | *F*-statistic *F*(1, 229) | Log. coefficients  beta, *p* |  |
| --- | --- | --- | --- | --- | --- |
| **DBTP** |  |  |  |  |  |
| **Past (1)** | -0.6692; 0.6751 | 0.8656 | 182.17, *p* < .001 | 3.4647, *p* < .0001 |  |
| **Future (1)** | -0.6756; 0.6543 | 0.6381 | 152.53, *p* <. 001 | 2.1615, *p* < .0001 |  |
| **Past (2)** | -06692; 0.6751 | 0.4983 | 182.17, p< .001 | 3.9983, *p* < .0001 |  |
| **Present (2)** | -0.7544; 0.7541 | 0.8447 | 253.20, *p* < .001 | 5.2173, *p* < .0001 |  |
| **Future (2)** | -0.6756;0.6543 | 0.2840 | 152.53, *p* < .001 | 2.7463, < .0001 |  |
| **SWB (3)** | -0.2981; 0.2610 | 0.0167 | 19.66, *p* < .001 | 0.0145, *p =* .9469 |  |
| **SWLS (3)** | -0.3879; 0.3617 | 1.0357 | 35.65, *p* < .001 | 0.81141, *p* < .0001 |  |
| **PHQ15 (4)** | 0.0932; -0.1259 | 1.0436 | 3.020, *p* = .0836 | -0.2402, *p* = .0846 |  |
| **SCSKD (5)** | 0.1132; 0.1206 | -0.2543 | 3.075, *p* = .0809 | -0.3835, *p =* .0241 |  |
| **PPQ (5)** | -0.5125; 0.4413 | 0.7187 | 65.686, *p* < .001 | 0.9484, *p* < .001 |  |
| **SBI (5)** | -0.4358; 0.3411 | 0.3679 | 38.845, *p* < .001 | 0.4659, *p =* .0162 |  |
| **RFS (5)** | -0.2866; 0.3067 | 0.3438 | 21.582, *p* < .001 | 0.5237, *p =* .0041 |  |
| **DBTP (6)** | 0.8114; -0.8119 | 1.491 | 338.10, *p* < .001 | -5.712, *p* < .001 |  |
| **SEM (7)** | NA | NA | NA | NA |  |
| **AGE (8)** | -0.0162; -0.0384 | 1.0033 | 0.029, *p* = .8660 | -0.0225, *p* = .8650 |  |

*Note:* Prevalence by Mediansplit. Parentheses: identical numbers indicate same model. LDF coefficients: imply relevance of feature variables; scaled in units of the discriminant value (metric LDF outcome).

*Table S7: Inferential Statistics of the Discriminant Coefficients, SEM modeled*

|  | Centroids  balanced [0; 1] | LDF coefficients | *F*-statistic *F*(1, 229) | Log. coefficients  beta, *p* |  |
| --- | --- | --- | --- | --- | --- |
| **SEM** |  |  |  |  |  |
| **Past (1)** | -0.6630; 0.6751 | 0.8673 | 179.74, *p* < .001 | 3.3276, *p* < .001 |  |
| **Future (1)** | -0.6695; 0.6543 | 0.6278 | 149.22, *p* < .001 | 2.006, *p* < .001 |  |
| **Past (2)** | -0.6630; 06751 | 0.4922 | 179.74, p< .001 | 3.9322; *p* < .0001 |  |
| **Present (2)** | -0.7533; 0.7541 | 0.8573 | 252.64, *p* < .001 | 5.2847, p < .001 |  |
| **Future (2)** | -0.6695; 0.6543 | 0.2712 | 149.22, *p* < .001 | 2.4863; *p* < .0001 |  |
| **SWB (3)** | -0.2996; 0.2610 | 0.0374 | 19.80, *p* < .001 | 0.031, *p =* .8885 |  |
| **SWLS (3)** | -0.3820; 0.3618 | 1.0169 | 34.87, *p* < .001 | 0.7874, *p* < .0004 |  |
| **PHQ15 (4)** | 0.079; -0.1259 | 1.0436 | 2.654, *p* = .1047 | -0.2253, *p* = .1050 |  |
| **SCSKD (5)** | 0.0959; 0.1206 | -0.2360 | 2.6561, *p* = .1045 | -0.3580, *p =* .0035 |  |
| **PPQ (5)** | -0.5137; 0.4413 | 0.7110 | 65.893, *p* < .001 | 0.9385, *p* < .001 |  |
| **SBI (5)** | -0.4416; 0.3411 | 0.3771 | 39.303, *p* < .001 | 0.4802, *p =* .0128 |  |
| **RFS (5)** | -0.2952; 0.3067 | 0.3517 | 22.452, *p* < .001 | 0.5358, *p =* .0036 |  |
| **DBTP (6)** | NA | NA | NA | NA |  |
| **SEM (7)** | -0.8061; 0.8051 | 1.4692 | 323.60, *p* < .001 | 5.019, *p* < .001 |  |
| **AGE (8)** | -0.0205; -0.0385 | 1.0022 | 0.019, *p* = .8920 | -0.0181, *p* = .8910 |  |

*Note:* Prevalence by Mediansplit. Parentheses: identical numbers indicate same model. LDF coefficients: imply relevance of feature variables; scaled in units of the discriminant value (metric LDF outcome).

*Table S8: Multivariate Statistics of the LDF Equations*

|  | *ᴦ* | *Wilks λ, χ^2^(df)* | *F-Statistic* | *AIC* | *ᴦ* | *Wilks λ, χ^2^ (df)* | *F-Statistic* | *AIC* |
| --- | --- | --- | --- | --- | --- | --- | --- | --- |
| ***Mediansplit*** | **DBTP** |  |  |  | **SEM** |  |  |  |
| **BTPS**  Past, Future | 0.5040 | λ = 0.4959 *χ*^2^(2) = 159.92 *p* < .001 | *F*(2, 228) = 116.38 *p* < .0001 | 130.86 | 0.4989 | λ = 0.5010 *χ*^2^(2) = 157.58 *p* < .001 |  | 135.33 |
| **mBTPS** Past, Present, Future | 0.5751 | λ = 0.4249 *χ*^2^ (3) = 194.72 *p* < .001 | *F*(3, 227) = 102.85 *p* < .0001 | 73.348 | 0.5726 | λ = 0.4274 *χ*^2^ (3) = 193.38 *p* < .001 |  | 75.96 |
| **Well-being** SWB, SWLS | 0.1342 | λ = 0.8658 *χ*^2^ (2) = 32.86 *p* < .001 | *F*(2, 228) = 17.75 *p* < .0001 | 292.96 | 0.1317 | λ = 0.8683 *χ*^2^ (2) = 32.20 *p* < .001 | *F*(2, 228) = 17.37 *p* < .0001 | 293.63 |
| **PHQ15 (4)** | 0.0129 | λ = 0.9870 *χ*^2^ (1) = 2.989 *ns* | *F*(1, 229) =3.020 *p* = .0836 | 321.17 | 0.0114 | λ = 0.9886 *χ*^2^ (1) = 2.619 *ns* | *F*(1, 229) = 2.654 *p* = .1047 | 321.54 |
| **Traits** SCSKD, PPQ, SBI, RFS | 0.2768 | λ = 0.7232 *χ*^2^ (4) = 73.56 *p* < .001 | *F*(4, 226) =21.72 *p* < .0001 | 252.30 | 0.2775 | λ = 0.7225 *χ*^2^ (4) = 73.78,  *p* < .001 | *F*(4, 226) =21.79 *p* < .0001 | 252.08 |
| **DBTP (6)** | .5952 | λ = 0.4048 χ^2^ (1) = 206.65 *p* < .001 | *F*(1, 229) =338.10 *p* < .0001 | 97.56 | NA | NA | NA | NA |
| **SEM (7)** | NA | NA | NA | NA | .5845 | λ = 0.4155 *χ*^2^ (1) = 200.69 *p* < .001 | *F*(1, 229) =323.60 *p* < .0001 | 107.90 |
| **AGE (8)** | 0.0001 | λ = **0.9999** *χ*^2^ (1) = 0.0223 *ns* | *F*(1, 229) = 0.029 *p* = .8660 | 324.17 | **0.00008** | λ = **0.9999** *χ*^2^ (1) = 0.0223 *ns* | *F*(1, 229) = 0.019 *p* = .8920 | 324.18 |
| **HCA2** | **DBTP** |  |  |  | **SEM** |  |  |  |
| **mBTPS** Past, Present, Future | 0.6014 | λ = 0.3986 *χ*^2^ (3) = 120.03 *p* < .001 | *F*(3, 132) = 66.87 *p* < .0001 | 45.873 | 0.6386 | λ = 0.3614 *χ*^2^ (3) = 132.82 *p* < .001 | *F*(3, 132) = 78.34 *p* < .0001 | 25.285 |
| **DBTP Coefficient** | 0.6397 | λ = 0.3603 χ^2^ (1) = 134.24 *p* < .001 | *F*(1, 134) = 239.64 *p* < .0001 | 42.45 | NA | NA | NA | NA |
| **SEM Coefficient** | NA | *NA* | NA | NA | 0.6131 | λ = 0.3869 *χ*^2^ (1) = 124.87 *p* < .001 | *F*(1, 134) = 252.90 *p* < .0001 | 36.35 |

*Note:* Prevalence by Mediansplit, for some models compared to binary clustering (HCA2). Gamma: Standardized eigenvalue of the LDF, expresses explained variance.

*Table S9: Area Under the Curve: Balancedness ~ SWLS + DBTP*

| AUC | n | n_pos | n_neg |
| --- | --- | --- | --- |
| 0.9181 | 231 | 120 | 111 |

| Optimal cutpoint | youden | acc | sensitivity | specificity | tp | fn | fp | tn |
| --- | --- | --- | --- | --- | --- | --- | --- | --- |
| 1 | 0.8363 | 0.9177 | 0.9083 | 0.9279 | 109 | 11 | 8 | 103 |

Predictor summary:

| Data | Min. | 5% | 1st Qu. | Median | Mean | 3rd Qu. | 95% | Max. | SD | NAs |
| --- | --- | --- | --- | --- | --- | --- | --- | --- | --- | --- |
| Overall | 0 | 0 | 0 | 1 | 0.50649351 | 1 | 1 | 1 | 0.5010435 | 0 |
| 1 | 0 | 0 | 0 | 0 | 0.07207207 | 0 | 1 | 1 | 0.2597800 | 0 |
| 2 | 0 | 0 | 1 | 1 | 0.90833333 | 1 | 1 | 1 | 0.2897647 | 0 |

*Table S10: Area Under the Curve: Balancedness ~ SWLS + SEM*

| AUC | n | n_pos | n_neg |
| --- | --- | --- | --- |
| 0.9274 | 231 | 122 | 109 |

| optimal cutpoint | youden | acc | sensitivity | specificity | tp | fn | fp | tn |
| --- | --- | --- | --- | --- | --- | --- | --- | --- |
| 1 | 0.8548 | 0.9264 | 0.9098 | 0.945 | 111 | 11 | 6 | 103 |

Predictor summary:

| Data | Min. | 5% | 1st Qu. | Median | Mean | 3rd Qu. | 95% | Max. | SD | NAs |
| --- | --- | --- | --- | --- | --- | --- | --- | --- | --- | --- |
| Overall | 0 | 0 | 0 | 1 | 0.50649351 | 1 | 1 | 1 | 0.5010435 | 0 |
| 1 | 0 | 0 | 0 | 0 | 0.05504587 | 0 | 0.6 | 1 | 0.2291232 | 0 |
| 2 | 0 | 0 | 1 | 1 | 0.90983607 | 1 | 1.0 | 1 | 0.2875976 | 0 |

*Figure S1: Procedure*


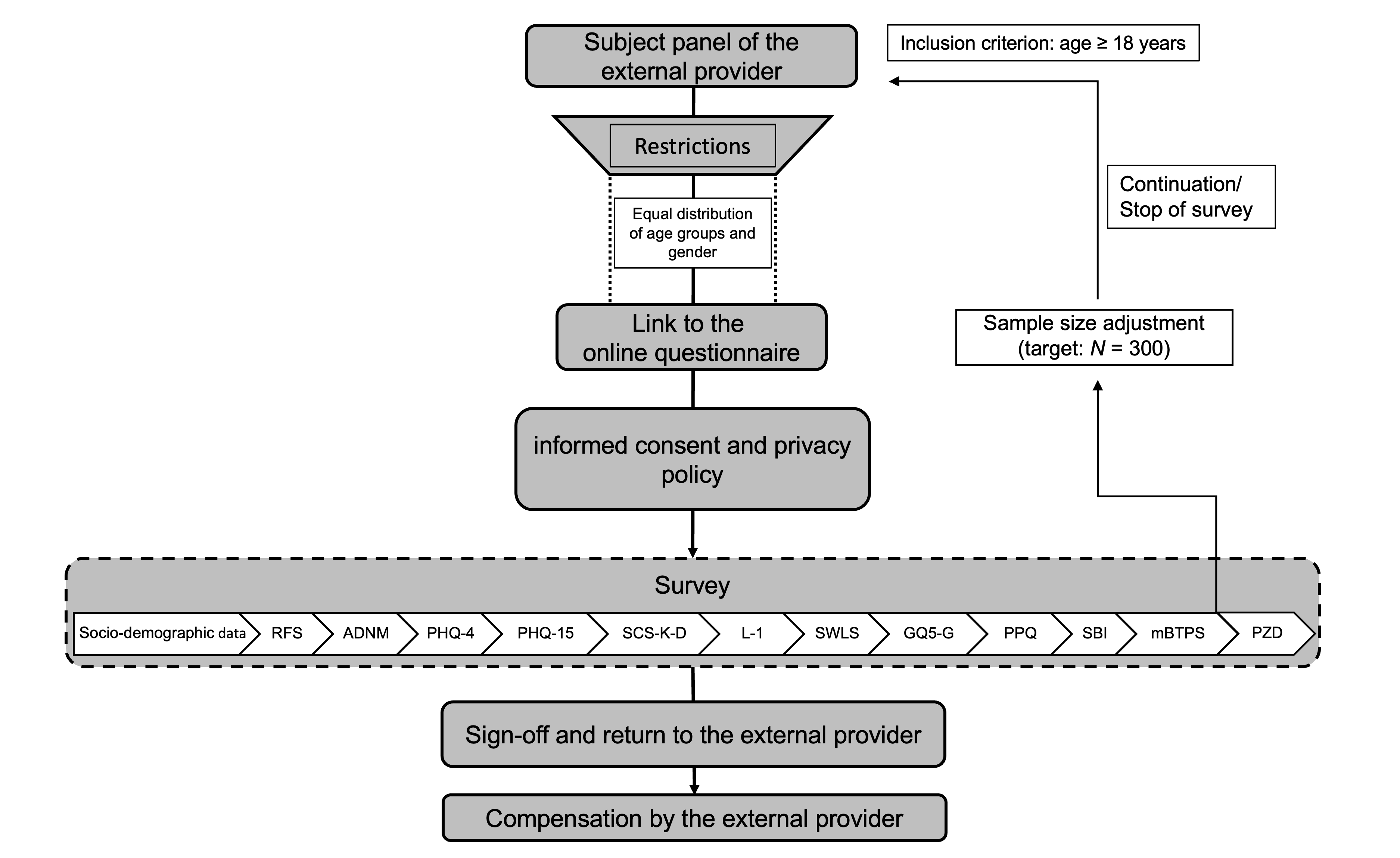


*Note*. RFS = Reminiscence Function Scale; ADNM = short form of the Adjustment Disorder New Model; PHQ-4 = Patient Health Questionnaire-4; PHQ-15 = Patient Health Questionnaire-15; SCS-K-D = short form of the Self-Control Scale; L-1 = one item scale to measure life satisfaction; SWLS = Satisfaction with Life Scale; GQ5-G = German adaptation of the Gratitude Questionnaire-Six Item Form; PPQ = Prioritizing Positivity Questionnaire; SBI = Savoring Beliefs Inventory; mBTPS = modified Balanced Time Perspective Scale; PZD = psychological temporal distance.

*Figure S2: BTP coefficient based on structural equation model (SEM)*


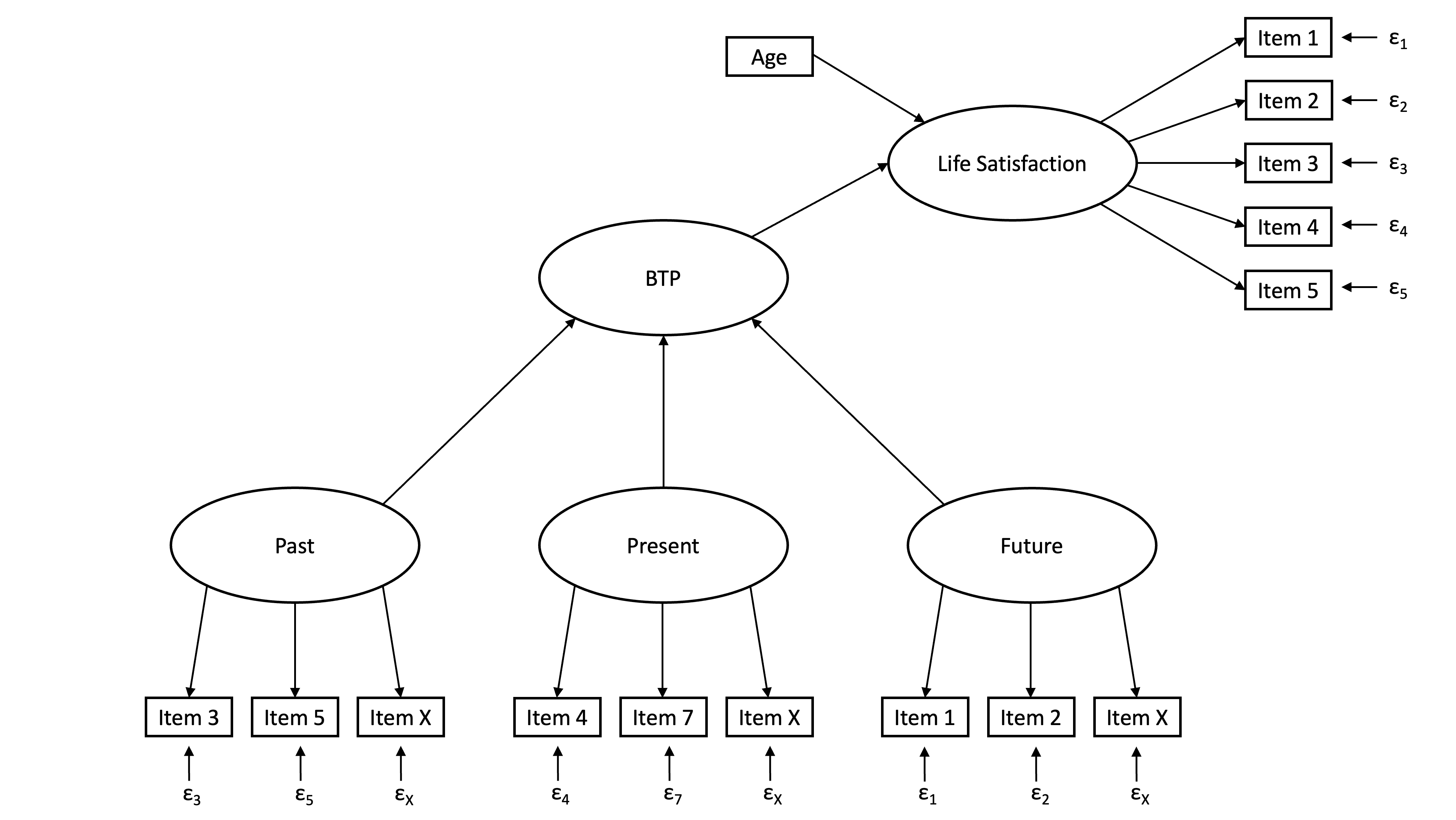


*Note:* BTP = Balanced Time Perspective, Life Satisfaction = SWLS.
The manifest variables (items) are first defined as indicators of the latent variables. In order to give the latent variables a metric, the loading of the first indicator was fixed at 1 in each case. In a second step (structural model), the relationship between BTP and life satisfaction was defined. The measure of BTP estimated based on this SEM was then extracted from the model and represents the SEM coefficient.
